# Nature-Inspired Superhydrophobic Coating Materials: Drawing Inspiration from Nature for Enhanced Functionality

**DOI:** 10.3390/mi15030391

**Published:** 2024-03-13

**Authors:** Subodh Barthwal, Surbhi Uniyal, Sumit Barthwal

**Affiliations:** 1 Department of Mechanical Engineering, Amity University, Greater Noida 201308, India; subodh_barthwal23@yahoo.co.in; 2 Department of Mechanical Engineering, Graphic Era University, Dehradun 248002, India; surbhiuniyal.87@gmail.com; 3 Nanomechatronics Lab, Kookmin University, Seoul 02707, Republic of Korea

**Keywords:** superhydrophobic, fabrication methods, wetting models, self-cleaning, coatings

## Abstract

Superhydrophobic surfaces, characterized by exceptional water repellency and self-cleaning properties, have gained significant attention for their diverse applications across industries. This review paper comprehensively explores the theoretical foundations, various fabrication methods, applications, and associated challenges of superhydrophobic surfaces. The theoretical section investigates the underlying principles, focusing on models such as Young’s equation, Wenzel and Cassie–Baxter states, and the dynamics of wetting. Various fabrication methods are explored, ranging from microstructuring and nanostructuring techniques to advanced material coatings, shedding light on the evolution of surface engineering. The extensive applications of superhydrophobic surfaces, spanning from self-cleaning technologies to oil–water separation, are systematically discussed, emphasizing their potential contributions to diverse fields such as healthcare, energy, and environmental protection. Despite their promising attributes, superhydrophobic surfaces also face significant challenges, including durability and scalability issues, environmental concerns, and limitations in achieving multifunctionality, which are discussed in this paper. By providing a comprehensive overview of the current state of superhydrophobic research, this review aims to guide future investigations and inspire innovations in the development and utilization of these fascinating surfaces.

## 1. Introduction

For many years, the interaction between surfaces and water has been a subject of keen interest. Nature provides numerous distinctive surface patterns designed to interact with water in ways that enhance functionality and adaptability to the environment. These natural designs serve as a foundational source for structural concepts extensively studied and implemented in the development of surfaces with specific water affinities. Various applications require surfaces with either high or low water affinity, underscoring the importance of investigating surface wettability and developing strategies to control it according to the desired outcome [1,2,3]. 

Wettability, a fundamental property describing the interaction between a solid surface and a liquid, is intricately linked to surface roughness and water contact angle. The water contact angle, a vital parameter quantifying wettability, reflects the degree of hydrophobicity, with higher angles denoting increased water resistance and lower surface energy. The terms “hydrophobic” and “hydrophilic” categorize materials based on their wetting behavior, describing materials as hydrophobic if they resist water and are challenging to wet and hydrophilic if they readily interact with water. These terms are derived from the Greek word “hydro”, meaning “water”, along with the suffixes “phobos” and “philia”, representing “fear” and “love”, respectively. Analyzing the static contact angle, which measures the angle formed by a static liquid droplet with the interphase of the solid surface and surrounding vapor, provides insight into the water–surface interaction. 

The water contact angle (WCA) is determined using a contact angle goniometer, providing insights into the interaction between a water droplet and a solid surface.

**Figure 1:**
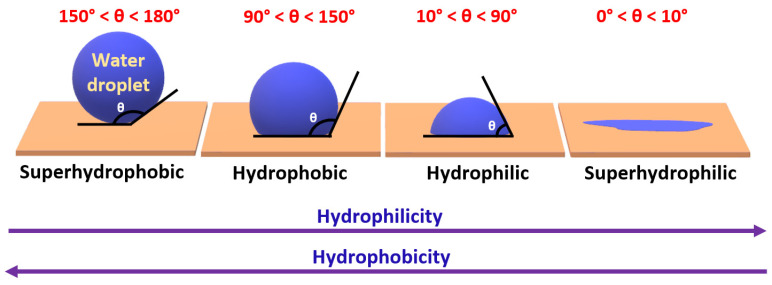
Diagram illustrating various surface wettability’s through contact angle measurements.

A material is categorized as hydrophilic when its water contact angle (WCA) is below 90°, while materials with a WCA exceeding 90° are termed hydrophobic. The term “superhydrophobic” is used to describe materials with a WCA ranging between 150° and 180°. “Superhydrophilic” surfaces feature a minimal contact angle, causing droplets to spread and completely wet the surface, while superhydrophobic surfaces showcase a maximal contact angle, resulting in droplets forming nearly spherical shapes, as illustrated in Figure 1. One of the remarkable features associated with superhydrophobic surfaces is their inherent self-cleaning ability. The combination of high water repellency and appropriate surface roughness allows water droplets to easily roll off the surface, carrying away contaminants and dust particles. This self-cleaning property has significant implications for various applications, ranging from anti-fouling surfaces in marine environments to low-maintenance coatings in architectural settings. Understanding the intricate relationships among surface roughness and surface chemistry is pivotal for advancing the design and application of functional surfaces with tailored wetting characteristics.

**Figure 2:**
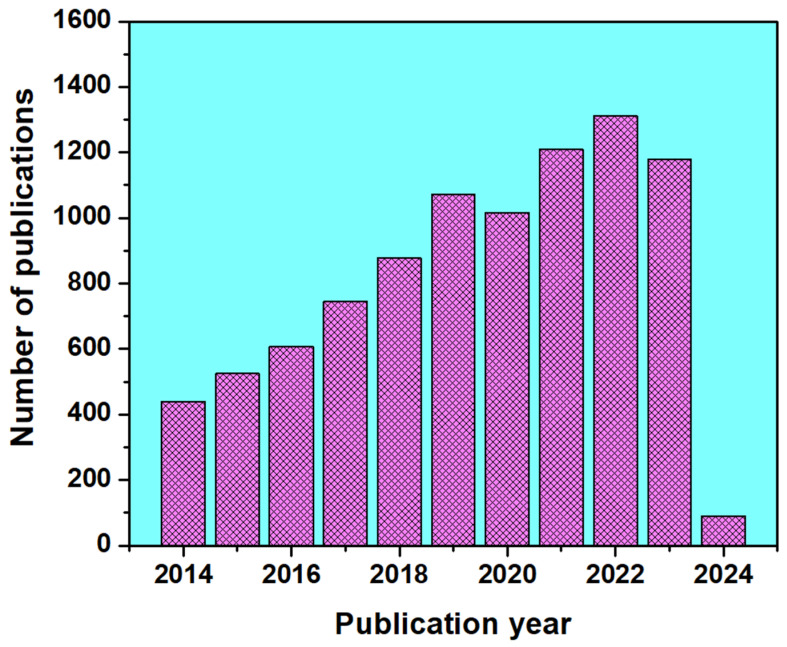
Statistics obtained from the Web of Science, revealing the total number of publications from 2014 to 2024 when using “superhydrophobic coating” as a keyword in the search.

The origins of superhydrophobic materials trace back to the early 20th century, where historical records document contact angles of 160° and above on coated surfaces treated with stearic acids and soot [4,5]. The fundamental principles of superhydrophobic behavior were subsequently established by Wenzel, Cassie, and Baxter in the ensuing decades. Although research progressed gradually from the 1940s to the 1990s, the crucial moment came in 1996 when T. Onda et al. validated artificial superhydrophobic surfaces [4,5,6,7,8,9,10]. The term “lotus effect” was officially patented by Neinhuis and Barthlott in 1997, referring to the naturally occurring superhydrophobic feature observed in lotus leaves [11]. This groundbreaking discovery ignited a surge in research, as evidenced by Chen et al.’s comprehensive review of superhydrophobicity in 1999 [12]. Figure 2 illustrates the steady increase in the development of “superhydrophobic coatings” over the past decade, based on the number of papers retrieved from the Web of Science. The notable rise in the number of publications in recent years highlights the growing interest and concern surrounding superhydrophobic coatings in our society.

**Figure 3:**
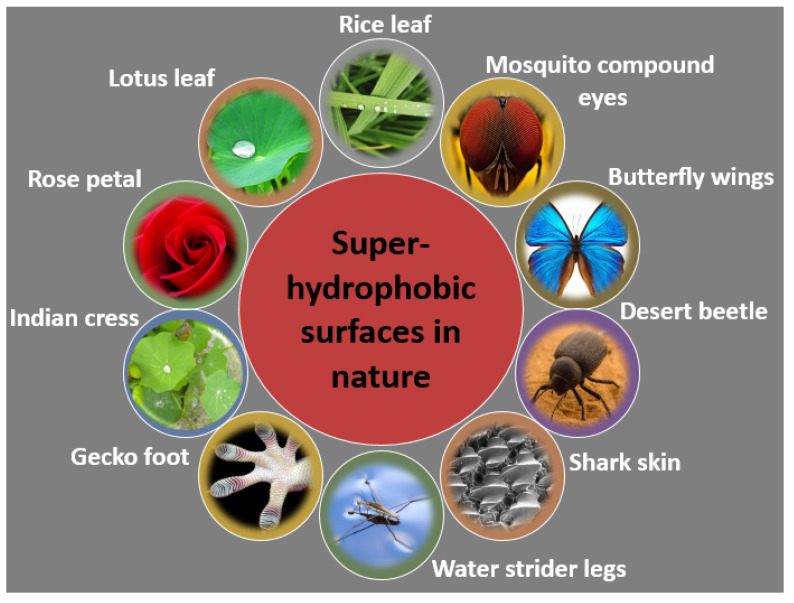
Superhydrophobic surfaces available in nature.

**Figure 4:**
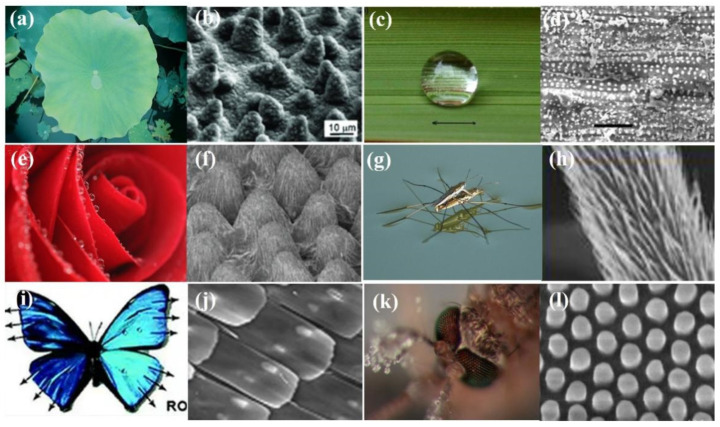
Diagram illustrating organisms with their SEM images: (**a**,**b**) lotus leaf; (**c**,**d**) rice leaf; (**e**,**f**) rose petal; (**g**,**h**) water strider; (**i**,**j**) butterfly wing; and a (**k**,**l**) mosquito compound eye [22].

Throughout the process of evolution, nature has ingeniously developed various biological systems with superhydrophobic surface properties, as shown in Figure 3. One prominent example of such surfaces is found in lotus leaves (*Nelumbo nucifera*), recognized for their exceptional superhydrophobicity. The discovery of the self-cleaning mechanism of lotus leaves can be attributed to botanists Barthlott and Ehler in 1977 [13]. Their investigation unveiled a sophisticated combination of hierarchical roughness, encompassing both microstructures and nanostructures, all covered beneath a layer of low surface energy wax. This unique combination of intricate roughness and a waxy coating contributes to the lotus leaves’ superhydrophobic nature, characterized by a water contact angle (WCA) of 161°. The synergy of high roughness and the presence of a waxy layer facilitates water droplets to effortlessly roll off the surface, effectively cleansing it by dislodging dust particles. This phenomenon is widely recognized as the “lotus effect”, as shown in Figure 4a,b.

Superhydrophobicity is not exclusive to lotus leaves but can be observed across various plants, insects, and birds, showcasing a diverse range of natural adaptations. Examples include rice (*Oryza sativa*) and taro (*Colocasia esculenta*) leaves, mosquito eyes, butterfly wings, desert beetles, gecko’s feet, water strider legs, and shark skins [14,15,16,17,18,19,20,21,22,23,24,25,26,27,28], as depicted in Table 1. Guo et al. [23] discovered that rice leaves possess remarkable superhydrophobicity, preventing water droplets from wetting their surface, as shown in Figure 4c. Their surface exhibits a binary microstructure and nanostructure similar to lotus leaves, featuring papillae with an average diameter of 5–8 mm in a one-dimensional order. The sublayer beneath the surface contains uniformly distributed nanometer-scale pins, enhancing air-trapping capability. This surface achieves superhydrophobicity with a WCA of 157.28°, facilitating the effortless rolling off of water droplets.

Similarly, an insightful study by Gao and Jiang [20] highlighted the remarkable superhydrophobic properties exhibited by water striders, focusing on their legs. The water striders’ ability to stand and glide swiftly on water is attributed to the complex combination of hierarchical microstructures and nanostructures. These structures include numerous oriented, tiny hairs known as microsetae, which possess fine nanogrooves. These needle-shaped setae exhibit diameters ranging from 3 microns down to several hundred nanometers, with an average length of about 50 µm and an inclined angle of approximately 20° from the leg’s surface, as illustrated in Figure 4g,h. This unique adaptation allows water striders to stand and move swiftly on the water’s surface.

Zheng et al. [17] observed that butterfly wings exhibit overlapping quadrate scales (150 mm in length and 70 mm in width), forming a periodic hierarchy in one direction (Figure 4i). Each scale’s surface features separate ridging stripes, consisting of fine nano-stripes with multi-layers of cuticle lamellae of different lengths and tilted slightly upward with emerging nano-tips. These distinctive structures create air pockets that significantly reduce the surface contact area with water on the wings, showcasing superhydrophobicity with a water contact angle (WCA) of 152°.

In recent years, several high-quality review articles in the superhydrophobic coatings field have summarized recent advancements, challenges, and applications [29,30,31,32,33,34,35,36,37,38,39]. Wang et al. [29] specifically focused on the development and mechanism of superhydrophobic and antimicrobial coatings, addressing potential applications and associated challenges in commercial adoption. Nomeir et al. [31] offered new perspectives on self-cleaning superhydrophobic coatings for solar energy applications, exploring the impact of dust accumulation on solar panel efficiency. Table 2 provides an overview of current reviews on bio-inspired superhydrophobic coatings across various applications.

This review delves into the physics of bio-inspired surfaces, relevant theories, and the fabrication of artificial superhydrophobic surfaces. Additionally, we discuss potential applications of superhydrophobic coatings along with current issues and challenges.

## 2. Theoretical Background of Wetting

**Figure 5:**
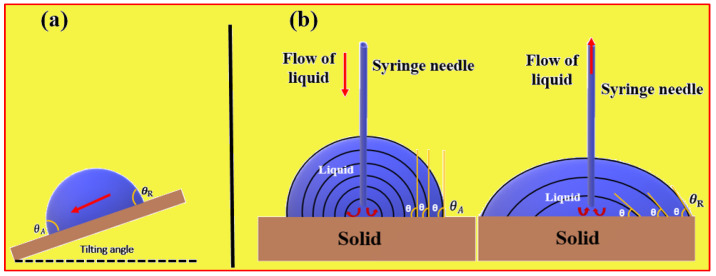
Measuring the advancing and receding angles by (**a**) tilting the substrate; and (**b**) changing the droplet volume by gradually introducing or extracting liquid from a sessile droplet.

Surface wettability is often assessed through the measurement of water contact angles (WCA), a widely accepted method for characterizing hydrophilic and hydrophobic surfaces. The contact angle is typically determined using contact angle goniometers, with a 2 µL or 5 µL water droplet positioned on the surface. Despite its reliability, variations in WCA readings can occur due to the heterogeneous nature of surfaces, both in terms of chemical composition and topography, leading to discrepancies of up to 20° on certain surfaces. Superhydrophobic surfaces, while often characterized by high static water contact angles (WCAs), may not always exhibit efficient water droplet roll-off. Some surfaces, like the petal surfaces of red roses, demonstrate a “pinning effect”, where water droplets remain spherical but do not easily roll off due to high adhesion forces [28]. Conversely, certain surfaces showcase a “slippery” behavior, challenging droplets to maintain stability. Therefore, beyond static contact angle, dynamic contact angle is also employed to characterize surface wettability. Contact angle hysteresis (CAH) is assessed through dynamic experiments involving droplets, providing insights into the adhesive properties between water droplets and the surface [40]. CAH (∆θ) is defined as the difference between the advancing contact angle (θ*_A_*) and the receding contact angle (θ*_R_*). θ*_R_*, indicating solid−liquid adhesion, is always smaller than or equal to $\theta_{A}$, representing solid–liquid cohesion. The advancing angle (*θ*_A_) and receding angle (*θ_R_*) are determined by tilting the substrate or changing the droplet volume. In the substrate tilting method, the solid surface is inclined at a specific angle. As the surface is tilted, the droplet on it begins to move, and the advancing contact angle ($\theta_{A}$) is measured when the droplet is in motion. Subsequently, the receding contact angle (θ*_R_*) is measured when the droplet either comes to rest or starts to retract, as shown in Figure 5a.

Alternatively, the droplet volume change technique involves intentionally altering the volume of the droplet, either by adding or removing liquid. As the droplet undergoes volume change, the contact angle also changes. *θ*_A_ is measured as the droplet increases in volume, and θ*_R_* is measured as the droplet decreases in volume, as shown in Figure 5b. Both techniques offer dynamic insights into the interaction between liquid droplets and solid surfaces, contributing to a comprehensive understanding of superhydrophobic and self-cleaning properties. Superhydrophobic surfaces with self-cleaning properties necessitate both high contact angles and low CAH. Advanced tools like contact angle goniometers, high-speed cameras, and image analysis software are often employed for precise measurement and analysis of the dynamic behavior of droplets on surfaces.

Various expressions of contact angle hysteresis are frequently discussed in the scientific literature, and it is crucial to make a clear distinction among them. A commonly employed experimental method for determining the contact angle of a droplet involves gradually increasing its volume until spreading initiates. The angle at which spreading commences, indicating that the droplet can overcome any energy barriers hindering its movement, is referred to as the advancing contact angle. Conversely, when the droplet volume is quasi-statically reduced, the contact line initially shifts at the receding contact angle. The disparity between the advancing and receding contact angles defines the contact angle hysteresis, which is zero for an ideal substrate but can be 10° or more for a real surface, posing a notorious challenge in measurement. This configuration is designated as unforced static hysteresis. Alternatively, it is feasible to exert force to propel a liquid drop across a surface and measure both the advancing angle at the front and the receding contact angle at the rear when the motion initiates. This scenario is denoted as forced, static hysteresis. It is crucial to highlight that the measured unforced and forced contact angle hysteresis for a specific drop on a particular surface may not necessarily align. This discrepancy arises because a forced drop undergoes deformation, and the free energy barriers are contingent on the shape of the drop.

Quantitatively understanding contact angle hysteresis poses challenges due to its dependence on the intricate characteristics of surface inhomogeneities, which are typically random in both position and size. Nevertheless, recent progress in surface engineering has enabled the creation of surfaces featuring well-defined chemical patterning with distinct areas exhibiting varying contact angles. 

Another noteworthy advancement involves the development of superhydrophobic surfaces. When surfaces with an inherent hydrophobic contact angle are covered with micron-scale posts, the macroscopic contact angle can, in specific cases, approach close to 180°. This innovation presents exciting possibilities for manipulating surface properties for various applications. Drops exhibit two distinct states on surfaces: a suspended state, known as the Cassie–Baxter state [41], where they rest on top of the posts, and a collapsed state, referred to as the Wenzel state [42], where they fill the interstices between the posts. Additionally, on these surfaces, drops can easily roll [43,44,45], highlighting the significance of contact angle hysteresis in comprehending this behavior. 

Kusumaatmaja and Yeomans [46] conducted an investigation into contact angle hysteresis on chemically patterned and superhydrophobic surfaces by quasi-statically altering the drop volume. Their study encompassed both two and three dimensions, employing analytical and numerical methods to minimize the free energy of the drop. In two dimensions, on a surface striped with regions exhibiting different equilibrium contact angles, *θ*, they observed a slip, jump, and stick motion of the contact line. The advancing and receding contact angles corresponded to the maximum and minimum values of *θ*, respectively. In three dimensions, these values served as bounds, with contact angle hysteresis being mitigated by the free energy associated with surface distortion [47,48]. Although stick, slip, and jump behavior persisted, it is important to note that defining a single macroscopic contact angle for patterns around the drop size becomes problematic. The position and magnitude of the contact line jumps proved to be sensitive to the details of surface patterning and could vary in different directions relative to that patterning.

On superhydrophobic surfaces, the behavior of drops in two dimensions reveals specific contact angles. In the ideal scenario, the advancing contact angle is 180°, as confirmed by previous studies [49]. Simultaneously, the receding angle corresponds to *θ_R_*, which represents the intrinsic contact angle of the surface. When considering collapsed drops in two dimensions, the advancing contact angle remains 180°. However, the receding angle is *θ_R_* −90° due to the necessity for the contact line to dewet the sides of the posts.

In the realm of three dimensions, both suspended and collapsed drops exhibit an advancing angle close to 180°, as supported by various sources [45,50,51]. However, there is an increase in the receding contact angle. Despite this increase, the receding angle remains notably smaller in the collapsed state, indicating stronger contact line pinning. Consequently, the hysteresis observed in suspended drops is generally much smaller than that observed in collapsed drops on the same surface.

**Figure 6:**
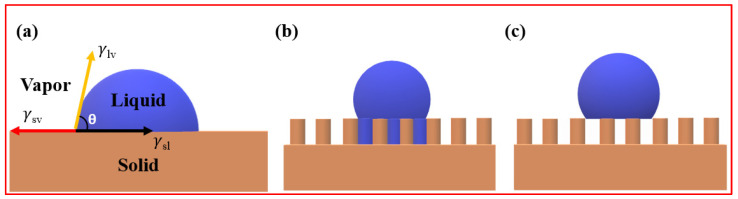
Schematics of wetting models: (**a**) Young model, (**b**) Wenzel model, and (**c**) Cassie–Baxter model.

Wettability is a phenomenon observed when water comes into contact with surfaces, wherein some surfaces readily attract water while others repel water droplets. This behavior is intricately influenced by the interplay of molecular, chemical, and physical forces occurring at the interface of liquid, solid, and gas phases. The interactions at the solid–liquid–air interfaces play a pivotal role in determining the behavior of water droplets on a surface. When a liquid encounters a surface, cohesive and adhesive forces come into play, dictating the shape of the liquid. If adhesive forces between the liquid and the surface are dominant over cohesive forces within the liquid, the liquid will spread, forming a thin film across the surface. Conversely, if cohesive forces within the liquid outweigh adhesion to the surface, a droplet will form, maintaining minimal contact with the surface. The theoretical foundation of wetting involves various models and principles designed to comprehend and predict these intricate interactions. Thomas Young, a British scientist in 1805, introduced the first comprehensive understanding of droplet-wetting characteristics on an ideally smooth, solid surface [52]. Young’s equation establishes a relationship between the contact angle (θ), surface tension (γ), and interfacial tensions among the three phases (solid, liquid, and gas) at the droplet’s contact point on a solid surface, as shown in Figure 6a.

According to Young’s equation, the contact angle of a liquid on a flat surface is given by:

(1) $$\cos\theta =\frac{\gamma_{sv-}\gamma_{sl}}{\gamma_{lv}}$$

where $\gamma_{sv}$, $\gamma_{sl}$, and $\gamma_{lv}$ refer to the interfacial tensions of the solid–vapor, solid–liquid, and liquid–vapor phases, respectively.

Despite its significance, Young’s equation has limitations, primarily being applicable only to surfaces with uniform chemical composition and smoothness. In reality, most surfaces exhibit an uneven chemical composition and varying degrees of roughness, rendering Young’s equation unsuitable in such scenarios. 

Therefore, the early work by Wenzel (1936) and later by Cassie and Baxter in 1944 has led to the development of two distinct models explaining wetting states on rough surfaces. 

Wenzel modified Young’s equation by introducing a roughness factor to elucidate wettability on textured surfaces [53]. In the Wenzel state, the liquid fully wets the textured surface, maximizing the liquid–solid contact area, as shown in Figure 6b. According to Wenzel’s theory, the apparent contact angle ($\theta_{a}$) of a liquid droplet placed on a rough, solid surface is given by the following equation:

(2) $$\cos\theta_{a}=r\cos\theta$$

Here, r represents the roughness factor, defined as the ratio of the actual contact area of the solid–liquid to the apparent contact area of the solid–liquid, and *θ* is the equilibrium contact angle of the liquid on a smooth surface of the same material.

Since r is always greater than one for a rough surface, Wenzel’s equation predicts that if (θ > 90°), ($\theta_{a}$ > θ), indicating a hydrophobic surface, and if (*θ* < 90°), ($\theta_{a}$ < θ), indicating a hydrophilic surface. Therefore, in the Wenzel state, the surface roughness will make intrinsically hydrophobic surfaces more hydrophobic and hydrophilic surfaces more hydrophilic. However, the equation has limitations and does not apply when the chemical composition of the solid surface differs. 

The Cassie–Baxter equation describes the wetting behavior of liquids on rough surfaces and is an extension of the Wenzel model. Proposed by Cassie and Baxter in 1944, this model accounts for the presence of air pockets trapped within surface asperities [54]. In the Cassie state, liquid droplets are supported by the tops of the rough features, and air is trapped underneath, creating a composite interface, as displayed in Figure 6c. The Cassie–Baxter equation is given by:

(3) $$\cos\theta^{*}=f_{1}\cos\theta -f_{2}$$

where $\theta^{*}$ is the apparent contact angle for a droplet on a rough surface; θ is the equilibrium contact angle obtained on an ideally flat surface of the same chemical composition; *f*_1_ is the fraction of solid substrate in contact with the liquid; and *f*_2_ is the fraction of air in contact with the liquid (i.e., *f*_1_ + *f*_2_ = 1). Thus, Equation (3) can be simplified into Equation (4):

(4) $$\cos\theta^{*}=f_{1}(\cos\theta +1)-1$$

If *f*_1_ is very small, it reveals there will be a large amount of air trapped under the water droplet, and $\cos\theta^{*}$ can approach −1 with the water apparent contact angle ($\theta^{*}$) becoming close to 180°. Hence, to achieve a high apparent contact angle, the practical contact area between the solid and liquid droplets should be as small as possible. 

The Cassie–Baxter state is characterized by enhanced liquid repellency due to the trapped air, preventing complete wetting of the surface. This model is particularly relevant for superhydrophobic surfaces, where the combination of surface roughness and air entrapment leads to extraordinary water-repellent properties. 

A set of equations was solved to characterize the dynamics and thermodynamics of the considered drops by using the lattice Boltzmann algorithm [46].

### 2.1. The Model Drop

The authors opted to describe the equilibrium properties of the drop using a continuum free energy model [55]:

(5) $$\Psi =\int_{V}\left(\psi_{b}\left(n\right)+\frac{K}{2\;}{\left(\partial_{\alpha }n\right)}^{2}\right)dV+\int_{S}\psi_{s}\left(n_{s}\right)dS.$$

ψ_b_(n) is a bulk free energy [31]:

(6) $$\psi_{b}\left(n\right)=p_{c\;}{\left(v_{n}+1\right)}^{2}(v_{n}^{2}-2v_{n}+3-2\beta \tau_{w})$$

where *υ_n_* = (*n* − *n_c_*)/*n_c_*, *τ_w_* = (*T_c_* − *T*)/*T_c_*, and *n*, *n_c_*, *T*, *T_c_*, and *p_c_* are the local density, critical density, local temperature, critical temperature, and critical pressure of the fluid, respectively.

In Equation (5), the second term represents the free energy attributed to interfaces within the system [55]. The final term in Equation (5) characterizes the interactions between the fluid and the solid surface. Cahn [56] defined the surface energy density as *ψ*_s_(*n*) = −ϕ*n_s_*, where *n_s_* represents the fluid density value at the surface. The parameter *ϕ* governs the strength of interaction, influencing the local equilibrium contact angle. The minimization of the free energy results in the establishment of the boundary condition at the surface:

(7) $$\partial_{⊥}n=-\phi /\kappa$$

and a relation between *ϕ* and the equilibrium contact angle *θ* [55]:

(8) $$\phi =2\beta \tau_{w}\sqrt{{2p}_{c}\kappa }\mathrm{sign}(\frac{\pi }{2}-\theta_{e})\;\sqrt{\cos\frac{\alpha }{3}(1-\cos\frac{\alpha }{3})}$$

where α = cos^−1^ (sin^2^ *θ*), and the function sign is employed to determine the sign of its argument. Equivalent boundary conditions can be applied to non-flat surfaces. A method for addressing corners and ridges essential for modeling superhydrophobic surfaces is outlined in [57]. The governing equations for the drop’s motion are the continuity and Navier–Stokes equations:

(9) $$\partial_{t}n+\partial_{\alpha }\left(nu_{\alpha }\right)=0$$

(10) $${\;\;\;\;\partial }_{t}(nu_{\alpha })+\partial_{\beta }(nu_{\alpha }u_{\beta })=-\partial_{\beta }P_{\alpha \beta }+\upsilon \partial_{\beta }[n{(\partial }_{\beta }u_{\alpha }+\partial_{\alpha }u_{\beta }+\delta_{\alpha \beta }\partial_{\gamma }u_{\gamma })]+na_{\alpha },$$

Here, *u*, *P*, *υ*, and a represent the local velocity, pressure tensor, kinematic viscosity, and acceleration, respectively. 

To simulate unforced static hysteresis, a gradual increase or decrease in drop volume is necessary. This is achieved by adjusting the drop liquid density by approximately ±0.1%. Consequently, the drop volume is influenced as the system returns to its coexisting equilibrium densities. For forced hysteresis, a body force *na_α_* is introduced into the Navier–Stokes Equation (6) to address the phenomenon.

### 2.2. Three-Dimensional Drop on a Chemically Patterned Surface

**Figure 7:**
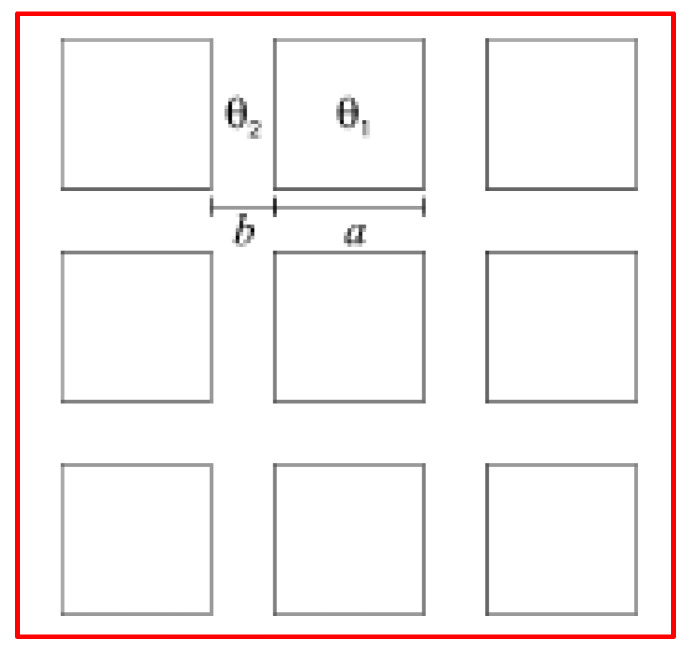
Figure of a chemically patterned surface [46].

Illustrated diagrammatically in Figure 7, the chemically patterned surface under consideration consists of squares with a side length *a* = 12, separated by a distance *b* = 5. In the first scenario (surface A), the squares’ equilibrium contact angle is *θ*_1_ = 110°, while that of the channels between them is *θ*_2_ = 60°. In the second case (surface B), exchange of the equilibrium contact angles takes place, i.e., *θ*_1_ = 60° for the squares and *θ*_2_ = 110° for the channels [46]. Despite the nearly identical macroscopic contact angle for both surfaces, *θ*_CB_ ≈ 85.5°, calculated using the Cassie–Baxter formula:

cos *θ*_CB_ = *f*_1_cos *θ*_1_ + *f*_2_cos *θ*_2_ (11)

which averages over the surface contact angles. The parameters *f*_1_ and f_2_ represent the fractions of the surface with intrinsic equilibrium contact angles *θ*_1_ and *θ*_2_, respectively. At first glance, one might anticipate the two surfaces to exhibit very similar behavior. However, this assumption proves incorrect unless the strength of the heterogeneities falls below a specific threshold [58,59]. This condition implies negligible contact angle hysteresis.

### 2.3. Three-Dimensional Suspended Drop on a Topologically Patterned Surface

The results from three-dimensional lattice Boltzmann simulations are presented in this section with the aim of investigating contact angle hysteresis on three-dimensional topologically patterned surfaces.

For the suspended state, we selected parameters *a* = 3, *b* = 7, l = 5, and *θ* = 110°. According to the Cassie–Baxter formula, this configuration provides an estimated macroscopic contact angle of *θ* = 160°. The extremely high value of the drop contact angle poses a challenge for simulations, as small changes in the contact angle necessitate significant adjustments in the drop volume. Additionally, to ensure the drop is suspended on an adequate number of posts, a substantial simulation box is required. Lattice with dimensions 168 × 168 × 168 is used, and the largest simulated drop had a volume of 16.8 × 10^5^.

The susceptibility of the drop shape near the surface (deviating from a spherical cap) to small variations can lead to substantial uncertainties in contact angle measurements. The equation used for that is:

(12) $$\theta {\mathrm{macro}}_{\;}=2\;\tan{-}^{1}(\frac{H}{r_{max}})$$

where *H* represents the height of the drop and *r_max_* is the maximum base radius, offering an alternative definition for the macroscopic contact angle.

**Figure 8:**
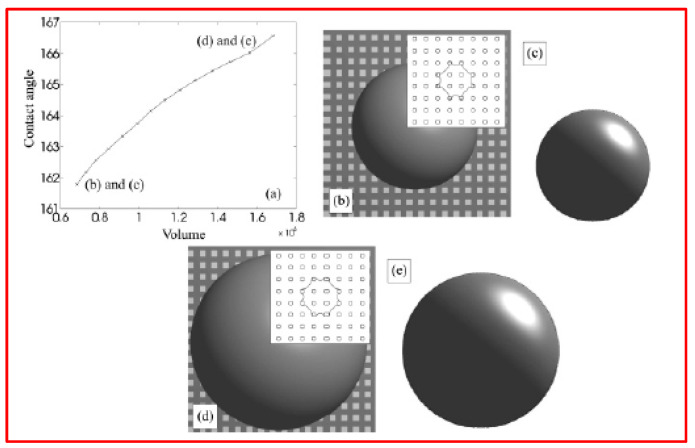
Contact line dynamics for a suspended drop on a topologically patterned surface. (**a**) Contact angle as a function of volume. (**b**,**c**) Top and side view of the drop at V = 6.8 × 10^5^. (**d**,**e**) Top and side view of the drop at 16.8 × 10^5^ [46].

Figure 8a illustrates the drop contact angle as a function of volume. In the simulation, we increased the volume from 6.8 × 10^5^ to 16.8 × 10^5^, resulting in a macroscopic contact angle *θ* (as per Equation (12)) ranging from 161.8 to 166.6. In Figure 8b–e, contour plots, top views, and side views of the drop at the beginning and end of the simulation are presented.

### 2.4. Three-Dimensional Collapsed Drop on a Topologically Patterned Surface

Now, we shift our focus to the collapsed state, where the space between the posts is filled with liquid. We chose the parameters *a* = 4, *b* = 6, *l* = 5, and *θ* = 120°, and according to the Wenzel formula, this configuration yields a contact angle *θ*_W_ = 154°.

**Figure 9:**
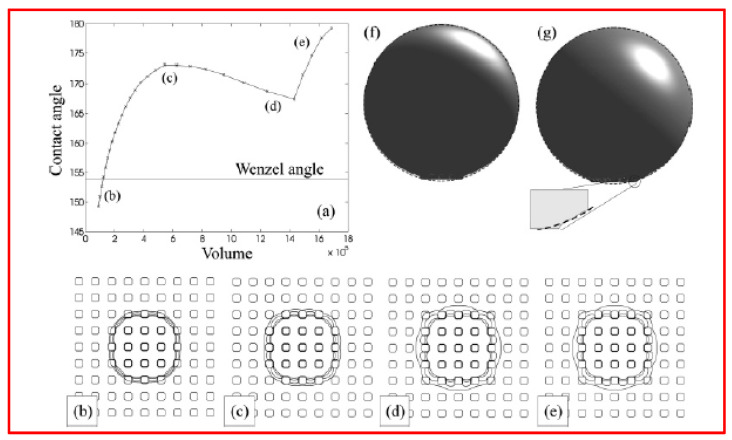
Contact line dynamics for a collapsed drop on a topologically patterned surface. (**a**) Contact angle as a function of volume. (**b**–**e**) Contour plots of the drop at positions indicated in panel (**a**). (**f**) The drop cross section in the horizontal direction. (**g**) The drop cross section in the diagonal direction [46].

Figure 9a depicts the macroscopic contact angle of the drop, as a function of increasing volume. Initially, the contact angle rises because, despite the contact line moving outward diagonally with respect to the posts, it remains pinned in the horizontal and vertical directions. This can be observed in the contour plots displayed in Figure 9b,c. Once the drop contacts the four neighboring posts along the diagonals, it wets the tops of these posts (Figure 9d,e), causing a slight decrease in the contact angle, approximately by 5°. At this stage, the contact line is pinned again until it becomes energetically favorable to jump and wet the neighboring posts in the vertical and horizontal directions. However, this contact line jump did not occur in the presented simulations due to the prohibitive computational cost of increasing the size of the simulation box.

In Figure 9f,g, typical side views of the drops are shown, with the dashed line representing the corresponding spherical fits. The fitted curves align well with drop profiles above the posts but not with profiles between the posts.

## 3. Fabrication of Superhydrophobic Surfaces 

**Figure 10:**
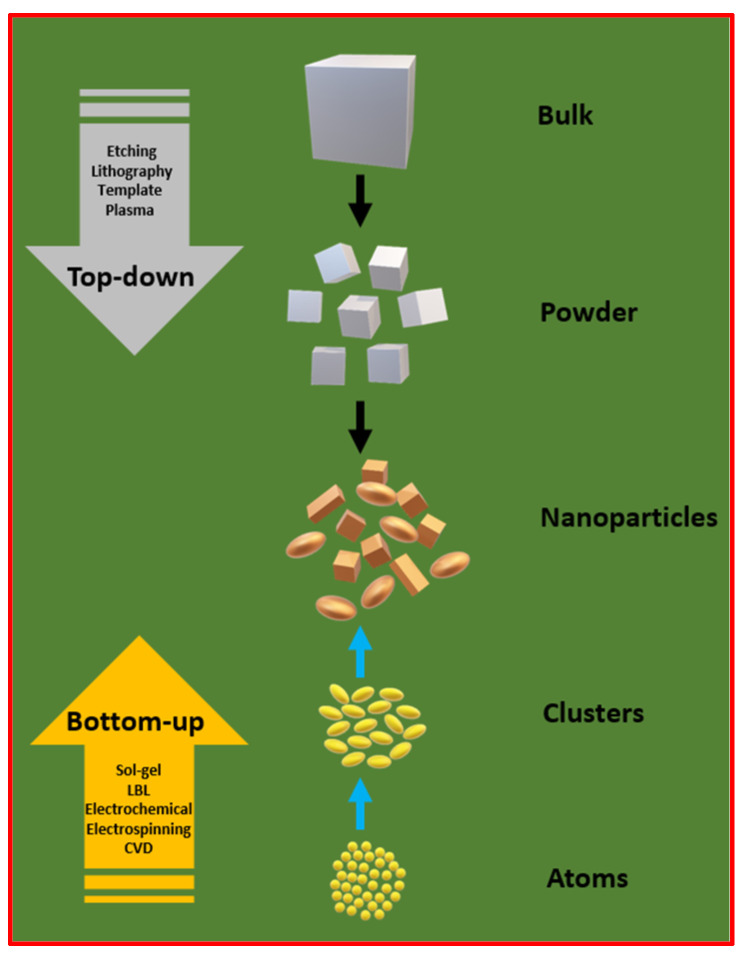
Diagram showing top-down and bottom-up methods for nanomaterial synthesis.

Artificial superhydrophobic surfaces, characterized by extremely high water repellency, can be engineered by precisely controlling surface roughness and surface energy—two critical parameters influencing solid surface wettability. Achieving this involves the creation of hierarchical micro/nanostructures on the surface and chemical modification using low-surface energy materials. Various methods are employed to achieve superhydrophobicity, often relying on two main approaches: top-down and bottom-up methods, as shown in Figure 10. Both approaches offer flexibility and can be combined to create intricate hierarchical structures. While top-down methods are often faster and more scalable, bottom-up methods provide enhanced control over material composition and can facilitate the incorporation of diverse functionalities. The selection between these approaches depends on the specific requirements of the application and the desired characteristics of the superhydrophobic surface.

### 3.1. Top-Down Synthesis 

Top-down synthesis for superhydrophobic surfaces involves the fabrication of such surfaces by modifying existing structures or materials at the macroscopic level to achieve the desired properties. This approach typically begins with a bulk material, and through various techniques such as etching, abrasion, or lithography, the surface is altered to create microstructures and nanostructures. The top-down synthesis approach provides precise control over the size, shape, and arrangement of nanostructures. Despite its advantages in precision and reproducibility, top-down synthesis may face challenges in terms of scalability and cost-effectiveness for large-scale production.

#### 3.1.1. Chemical Etching

Chemical etching stands out as a highly effective method for crafting superhydrophobic surfaces, utilizing a controlled chemical reaction to selectively remove material and engineer intricate microstructures and nanostructures on a substrate. In the superhydrophobic context, this process begins with the application of a specific etchant solution that interacts with the material surface (almost any metal or alloy), inducing reactions that result in the creation of tailored surface textures. The choice of etchant (strong acids, such as HCl and H_2_SO_4_, and a strong base, such as NaOH), along with parameters like concentration and duration, plays a pivotal role in shaping the final surface morphology. Chemical etching offers several advantages, including cost-effectiveness, scalability, and applicability to a diverse range of materials, making it a versatile option for creating superhydrophobic surfaces. 

**Figure 11:**
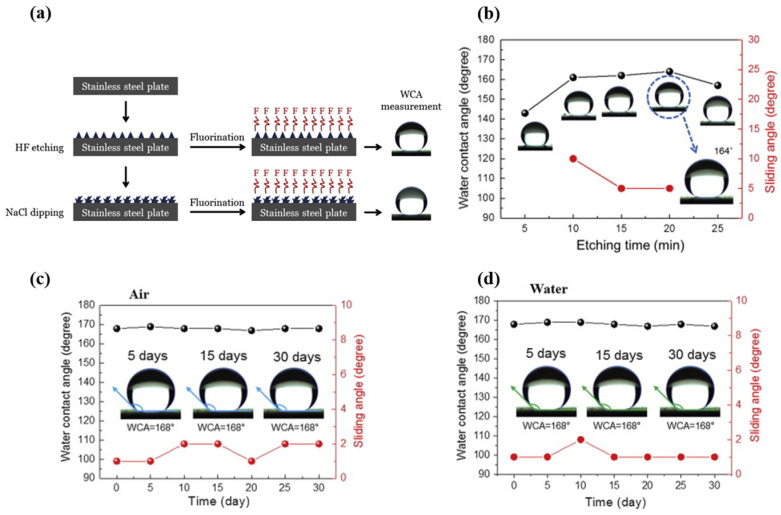
(**a**) Schematic depiction outlining the various steps involved in preparing superhydrophobic stainless steel surfaces; (**b**) changes in the WCA and SA of stainless steel following different etching durations in HF; durability assessment of the superhydrophobic stainless steel sample after exposure to (**c**) air and (**d**) water for a period of 1 month [61].

The success of chemical etching in fabricating superhydrophobic surfaces lies in its ability to generate hierarchical structures that replicate natural water-repellent surfaces found in certain plant leaves and animal skins. The resulting surface roughness, coupled with low surface energy coatings, leads to enhanced water repellency and low adhesion. This approach finds diverse applications, ranging from self-cleaning materials to anti-icing coatings, demonstrating the versatility and potential of chemical etching as a top-down method for precisely tailoring surface properties at the microscale and nanoscale [60,61,62,63,64,65]. For example, Kumar et al. [60] developed a robust and self-cleaning superhydrophobic coating for aluminum surfaces via a chemical etching method with a mixture of hydrochloric and nitric acids, followed by treatment with hexadecyltrimethoxysilane (HDTMS). The generated surface with rough rectangular pit-like microstructures displayed a WCA of 162.0 ± 4.2° and a sliding angle of 4 ± 0.5°. The coating displayed excellent thermal, chemical, and mechanical stability. Kim et al. [61] used a hydrogen fluoride (HF) solution to prepare superhydrophobic surfaces from austenitic stainless steel using a simple two-step chemical etching process. After fluorination, the surface showed a WCA of 166° and a SA of 5°. To further enhance the superhydrophobicity, the prepared stainless steel surface was dipped in a 0.1 wt.% NaCl solution at 100 °C, and the WCA was increased to 168° and the sliding angle was decreased to ∼2°. The prepared surface displayed self-cleaning properties with excellent superhydrophobicity after 1 month, as shown in Figure 11. 

**Figure 12:**
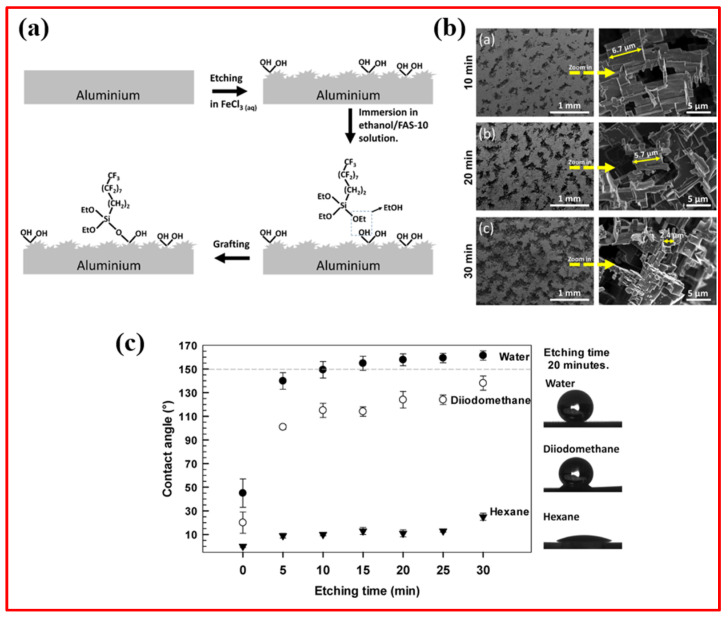
(**a**) Schematic diagram of the fabrication process of a superhydrophobic aluminum surface, including etching in a FeCl_3_ solution and subsequent modification with FAS-10; (**b**) surface morphologies of the surface etched for varying time intervals; and (**c**) contact angles measured for water, diiodomethane, and hexane on aluminum surfaces etched for different durations and treated for 30 min with 1 wt.% ethanol FAS-10 solutions [63].

Similarly, Zhu et al. [62] fabricated superhydrophobic magnesium alloy substrates by employing a one-step etching process and fluoride modification. The prepared magnesium surface with multiple hierarchical micro-nano structures displayed a water contact angle (WCA) of 173.3° and a sliding angle (SA) of 1°. Furthermore, the surface exhibited excellent properties, including self-cleaning, chemical stability even when exposed to air, chemically aggressive solutions (including strong acids and bases), and cyclic icing/melting treatments. Rodič et al. [63] outlined a process for producing superhydrophobic films on aluminum surfaces through a two-step method. This involved an initial etching step in a FeCl_3_ solution to create a hierarchical micro/nano-structured aluminum surface. Subsequently, grafting was carried out directly in an ethanol solution of 1H,1H,2H,2H-perfluorodecyltriethoxysilane (FAS-10) as a low-surface-energy material at ambient temperature, as displayed in Figure 12a,b. Optimizing the etching time to 20 min and the grafting duration to 30 min in FAS-10 solution yielded a superhydrophobic aluminum surface with a water contact angle exceeding 150° and a minimal sliding angle below 10°, as shown in Figure 12c. This superhydrophobic aluminum surface demonstrated effective self-cleaning against solid pollutants and enhanced anti-icing performance with delayed melting.

#### 3.1.2. Lithography Technique

Lithography emerges as a sophisticated and precise technique for generating superhydrophobic surfaces, relying on the principles of light and pattern transfer to create microstructures and nanostructures on a substrate. In lithography, a photosensitive material is exposed to light through a mask or a photomask, which carries the desired pattern. The exposed material undergoes chemical or physical changes, creating a positive or negative replica of the pattern on the substrate. The resulting surfaces are characterized by intricate patterns that effectively trap air pockets, reducing the contact area between water droplets and the surface. This, in turn, leads to the manifestation of superhydrophobicity.

The lithography-based technique can be categorized into various forms depending on the type of light source utilized. These include photolithography with ultraviolet (UV) and X-ray sources [66,67], nanoimprinting [68], electron beam lithography (EBL) [69], nanosphere lithography (NSL) [70], and laser lithography [71]. Photolithography, one of the primary forms of lithography, provides exceptional control over surface structures, allowing for the meticulous design of features at the microscale and nanoscale. This technique offers high resolution, excellent reproducibility, and scalability, making it an invaluable tool for tailoring surfaces to exhibit superhydrophobic properties. Nevertheless, ensuring a clean and smooth initial surface is crucial and potentially requires the use of a cleanroom.

Nanoimprint lithography involves pressing a mold with nanoscale features onto a material surface, transferring the pattern, and creating microstructures and nanostructures. The versatility of lithography makes it applicable to various substrates, and when combined with post-processing steps or additional coatings, it enhances the durability and multifunctionality of superhydrophobic surfaces. Lithography’s precision and versatility make it a crucial tool in the development of advanced materials for applications ranging from self-cleaning surfaces to microfluidic devices [72,73,74]. Ghasemlou et al. [72] prepared robust superhydrophobic surfaces featuring engineered lotus leaf-mimetic multiscale hierarchical structures through a hybrid approach that combines soft imprinting and spin-coating. The process involved direct soft-imprinting lithography onto starch/polyhydroxyurethane/cellulose nanocrystal (SPC) films, creating micro-scaled features resembling lotus leaves. Subsequently, low-surface-energy poly(dimethylsiloxane) (PDMS) was spin-coated over these microstructures. Further enhancement of the PDMS@SPC film involved modification with vinyltriethoxysilane (VTES) functional silica nanoparticles (V-SNPs), resulting in the fabrication of a superhydrophobic interface with a WCA of approximately 150° and a SA of less than 10°. The outcomes demonstrated that the prepared coating displayed outstanding moisture barrier properties, self-cleaning capabilities, and superior mechanical durability against various tests, including knife scratches, finger-rubbing, jet water impact, a sandpaper abrasion test for 20 cycles, and a tape-peeling test for approximately 10 repetitions.

Guo et al. [73] developed a flexible superhydrophobic microarray utilizing photolithography technology, achieving a maximum WCA of 151.1 ± 0.9° through the optimization of microcolumn spacing and height. To enhance its functionality, carbon black nanoparticles were incorporated into the superhydrophobic microarray, imparting outstanding photothermal conversion performance. This prepared microarray found applications in anti-icing and deicing, significantly delaying the complete freezing of a water droplet by 87%. Moreover, it demonstrated efficiency by requiring 43.1% less time for the removal of an ice pellet and 28.5% less time for an ice sheet. Wang et al. [74] presented a one-step approach for creating a durable superhydrophobic surface on stainless steel using direct ultrafast laser microprocessing. The laser texturing method generated dense hierarchical micro/nanostructures on the stainless steel surface, showcasing commendable thermal stability and excellent anti-icing performance.

#### 3.1.3. Template Method

The template method has emerged as a simple and versatile approach for crafting superhydrophobic surfaces by utilizing predefined templates or molds to impart well-defined microstructures and nanostructures onto a substrate. This methodology involves the replication of surface features found in nature or artificially designed templates onto a chosen material, creating intricate patterns that contribute to superhydrophobic characteristics. The template method can be outlined in three distinct steps: template creation, molding, and demolding. Natural templates, such as lotus leaves, or synthetic structures, serve as blueprints for the desired surface morphology. The substrate, often composed of polymers or metals, is then shaped to mimic the surface features of the template [75,76,77,78,79,80].

This approach is cost-effective, scalable, and reproducible, making it a widely adopted method for producing polymeric surfaces. Additionally, the template method enables the incorporation of various materials and functionalities, providing flexibility in the design of surfaces with multifunctional properties. As a result, the template method emerges as a promising avenue for fabricating customized superhydrophobic surfaces tailored to specific industrial and technological applications.

**Figure 13:**
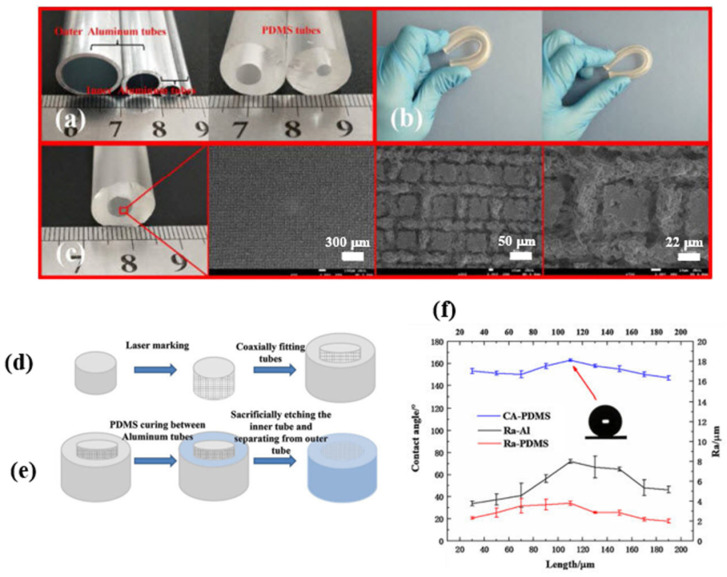
Prepared (**a**) various sizes of templates and PDMS tubes; (**b**) flexibility assessment of the PDMS tubes; (**c**) SEM images of the flexible tubes with corresponding scales of 300 μm, 50 μm, and 22 μm, respectively. The flowchart outlines the preparation of flexible tubes: (**d**) template preparation; (**e**) superhydrophobic flexible tube fabrication; (**f**) impact of processing spacing on roughness (Ra) and contact angle [77].

Wang et al. [75] manufactured a superhydrophobic fluororubber surface featuring a systematically layered microprotrusion structure by directly replicating the surface microstructure of stainless steel mesh. In the process, the treated stainless steel mesh was positioned onto a fluororubber sheet, and following vulcanization, the mesh was carefully removed. The resulting superhydrophobic fluororubber surface showcased a well-defined concave–protrusion structure, demonstrating outstanding superhydrophobicity with a contact angle of 153.93 ± 0.39°. Additionally, the fabricated surface exhibited remarkable thermostability and mechanical durability, enhancing its potential for practical applications. Xu et al. [76] showcased an efficient method for producing superhydrophobic PDMS films with low adhesion, employing a chemically etched template and subsequent thermal curing. The chemically etched nickel template was replicated to attain a large-scale superhydrophobic PDMS film with micro/nanostructures through a roll-to-roll thermal curing process. The cured PDMS film not only exhibited impressive liquid-repelling and self-cleaning characteristics but also demonstrated high transparency, along with robust chemical and mechanical durability. Wang et al. [77] generated hydrophobic templates with varied wettability by adjusting the laser etching distance on the surface of 6061 aluminum (Al) alloy tubes. Afterward, superhydrophobic flexible tubes were prepared, utilizing the prepared Al alloy tubes as templates and polydimethylsiloxane (PDMS) for replication, resulting in a water contact angle (WCA) of 162.8°. Remarkably, the superhydrophobic tubes exhibited exceptional durability and resistance to abrasion, and the prepared surface also demonstrated blood repellency, as shown in Figure 13.

#### 3.1.4. Plasma Treatment 

The plasma method represents an effective approach for fabricating superhydrophobic surfaces, leveraging the controlled modification of surface properties through plasma treatment. In this technique, a substrate material is subjected to a plasma discharge, inducing various chemical and physical changes on its surface. The controlled exposure to plasma allows for the creation of microstructures and nanostructures, contributing to the development of superhydrophobic characteristics. Plasma-based surface modification majorly involves plasma etching, plasma sputtering, and plasma polymerization [81]. 

Plasma etching, also known as dry etching, is a simple method for modifying the surface properties. During plasma etching, a low-pressure gas is introduced into a chamber, and an electric field is applied to ionize the gas, forming a plasma. This plasma contains energetic ions and electrons that react with the material’s surface. The specific chemistry of the gas, along with the parameters of the plasma (such as power, pressure, and temperature), determines the etching characteristics. This fabrication method can alter the wettability of the substrate by increasing roughness [82] or changing the functional group of the surface [83,84,85,86]. For example, Somrang et al. [86] reported the fabrication of nanostructured patterns through CF4 plasma etching on SiO_2_-based substrates, followed by treatment with Teflon coatings to achieve superhydrophobicity and antireflection. Initially, nickel-thin films were deposited on the substrates using the PVD sputtering process with varying deposition times. To promote the agglomeration of nickel nanoparticulate patterns, serving as etch-resistant masks, the thin films underwent rapid thermal annealing at 500 °C. These patterned substrates were subsequently subjected to CF_4_ plasma etching, followed by nickel mask removal through a nitric acid rinse. Finally, polytetrafluoroethylene films were applied to the nanostructured surface to achieve superhydrophobicity. 

Plasma sputtering, or physical vapor deposition (PVD), is a highly precise method used to deposit thin films onto surfaces. In this process, a high-energy plasma is generated in a vacuum chamber, and a target material is bombarded with energetic ions from the plasma. These ions dislodge atoms from the target material, creating a vapor that condenses on the substrate, forming a thin, uniform film. Becker et al. [87] introduced a method utilizing laser-assisted magnetron sputtering as a smart approach to achieve superhydrophobic properties on a poly(ethylene terephthalate) (PET) substrate. This innovative technique enables the creation of hierarchical surfaces with precise control. In this process, plasma sputter-deposited fluoropolymers are applied to PET substrates using the Nd/YAG laser-assisted magnetron sputtering technique, employing a poly(tetrafluoroethylene) (PTFE) target.

Similarly, plasma polymerization is a versatile technique for depositing thin polymer films on various substrates using plasma-enhanced chemical vapor deposition (PECVD). During this process, a monomer gas is introduced into a vacuum chamber containing plasma. The plasma breaks down the monomer molecules, initiating polymerization on the substrate surface. Plasma polymerization is utilized in applications ranging from biomedical coatings to surface modifications due to its ability to create uniform and adherent polymer layers on diverse materials [88,89,90]. Siddig et al. [90] successfully created a robust flame-resistant and highly hydrophobic phosphorus–fluoride coating on aramid fabrics using plasma-induced graft polymerization. Initially, the aramid fabrics underwent activation and roughening through low-pressure plasma treatment. Subsequently, a phosphorus–fluoride emulsion copolymer mixture was sequentially applied as a coating. In flame tests, the fabric demonstrated remarkable resilience with a char length of only 0.68 cm, showing no observable after-flame or after-glow times. 

**Figure 14:**
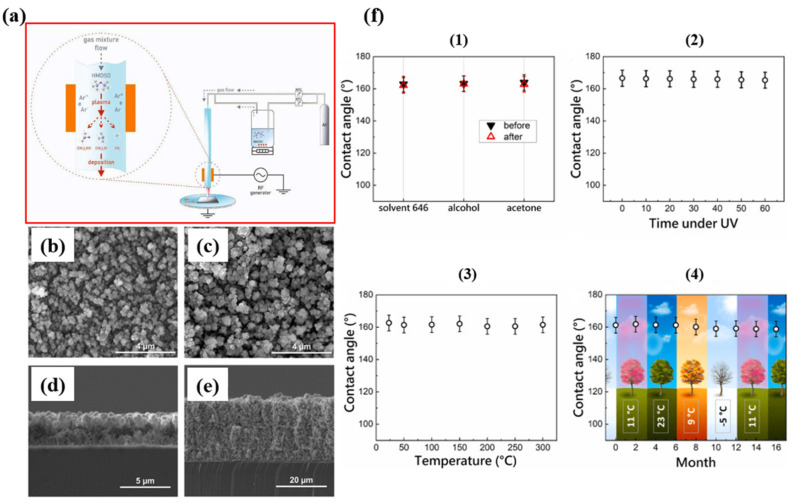
(**a**) Experimental setup schematic for superhydrophobic film deposition via atmospheric pressure plasma polymerization. SEM images depicting the film surface on a silicon substrate after one (**b**) and ten (**c**) cycles of plasma polymerization. Cross-sectional views of thin films after one (**d**) and ten (**e**) cycles. Image (**f**) displays contact angle graphs obtained from chemical tests (1), UV tests (2), thermal tests (3), and over time (4) [91].

Ussenkhan et al. [91] demonstrated the successful production of superhydrophobic films using the hexamethyldisiloxane (HMDSO) plasma polymerization method in an RF discharge plasma jet at atmospheric pressure. These films were applied using 3D printing technology, enabling large-scale application of the superhydrophobic coating, as shown in Figure 14. Their study revealed that the contact angle of the films was directly influenced by the RF discharge power, with HMDSO concentration and the number of cycles having no effect on the superhydrophobic properties but impacting optical properties, leading to reduced light transmission. While the morphology of the films remained consistent at one and ten deposition cycles on silicon substrates, there was a significant difference in film thickness. Films deposited in one cycle had a thickness of approximately 3 μm, whereas those deposited in ten cycles measured around 24.5 μm, as depicted in Figure 14b–e. This suggests that increasing the number of deposition cycles results in thicker films, affecting optical transparency. Under optimal conditions, the films achieved a contact angle of approximately 165°, indicating a highly hydrophobic surface. When applied as a single layer, the transmittance at 700 nm was about 88%, compared to 92% for pure glass. Surface morphology studies revealed that the plasma jet created microstructured and nanostructured coatings with high surface roughness, imparting superhydrophobic properties to the surface. Additionally, the resulting superhydrophobic films demonstrated stability and resistance to chemical solutions, high temperatures, UV irradiation, and weathering, as shown in Figure 14f.

### 3.2. Bottom-Up Synthesis 

Bottom-up synthesis is a method employed for the preparation of superhydrophobic surfaces that involves building up nanostructures or materials from smaller components or molecules. In contrast to top-down approaches, which start with larger structures and reduce them, bottom-up synthesis starts at the molecular or nanoscale level and assembles these components to create the desired structures. This technique often relies on self-assembly processes driven by molecular interactions, such as van der Waals forces, hydrogen bonding, or chemical reactions. By carefully designing and manipulating these interactions, researchers can create nanostructures with specific properties that contribute to superhydrophobicity. Bottom-up approaches are advantageous for their ability to produce intricate and precisely controlled nanostructures, providing a high level of customization for superhydrophobic surface properties. Common bottom-up techniques for superhydrophobic surface preparation include hydrothermal techniques, chemical vapor deposition, sol–gel processes, electrochemical deposition, and layer-by-layer deposition. 

#### 3.2.1. Sol–Gel Method

The sol–gel method is a versatile and widely used technique for fabricating superhydrophobic surfaces, offering control over surface properties and morphology. In this process, a sol, which is a colloidal suspension of nanoparticles, is formed by the hydrolysis and condensation of precursor compounds such as metal alkoxides or organometallic inorganic salts. The sol is then applied to a substrate, and subsequent gelation leads to the formation of a three-dimensional network. The resulting gel undergoes a drying and curing process to produce a solid film on the substrate.

To achieve superhydrophobicity, various modifications can be introduced during or after the sol–gel process. Incorporating hydrophobic agents such as silanes or fluorinated compounds enhances the water-repellency of the surface. Additionally, creating micro/nanostructures within the sol–gel matrix further contributes to the superhydrophobic characteristics by reducing surface energy and promoting the Cassie–Baxter state, where water droplets rest on the surface with minimal contact [92,93,94,95].

The sol–gel method stands out for its simplicity, cost-effectiveness, and scalability. It has been applied to diverse substrates, including glass, metal, and polymers, making it a promising approach for developing superhydrophobic surfaces with tailored functionalities for applications in self-cleaning, anti-fouling, and corrosion resistance. However, this method exhibits several drawbacks, such as the fact that the process can be time-consuming, particularly for thick coatings, and achieving precise control over microstructure and purity is challenging, affecting reproducibility. Compatibility issues with certain substrates and the potential for shrinkage and cracking during drying are additional limitations. Liang et al. [96] synthesized superhydrophobic silica powder through the sol–gel process and incorporated it into PDMS to formulate a transparent and resilient SiO_2_/PDMS composite coating. The use of the PDMS polymer aimed to improve the adhesion between the coating and the substrate, enhancing thermal, chemical, and mechanical stability. The spin-coated glass surface exhibited a transmittance exceeding 80% and a WCA of 158°. The resulting SiO_2_/PDMS composite coating maintained its superhydrophobicity even under challenging environmental conditions, including varying temperatures and exposure to strong chemicals.

**Figure 15:**
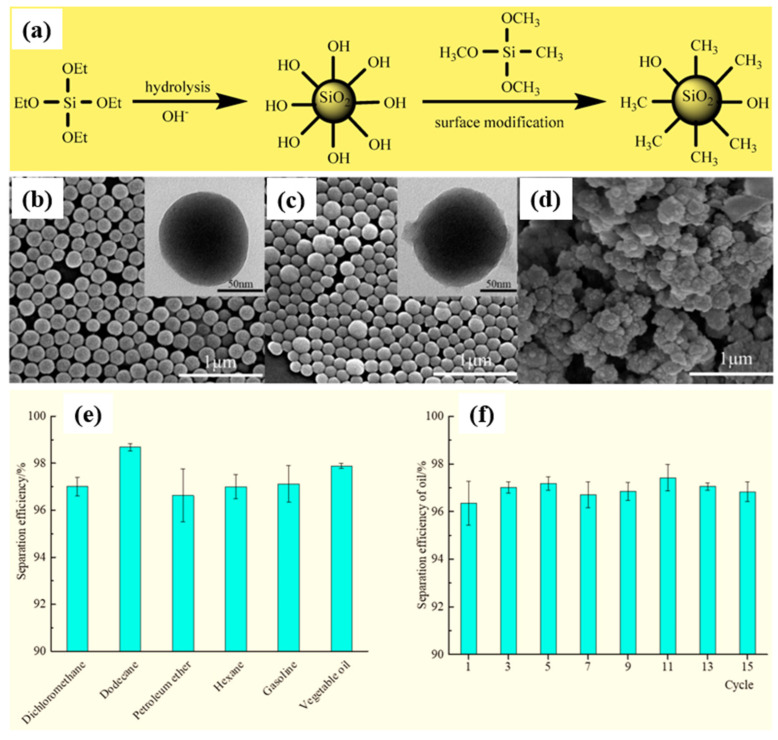
(**a**) Schematic of the preparation of the methylated silica sol; SEM and TEM images of (**b**) pristine silica; (**c**) methylated silica; and (**d**) methylated silica coating. Separation efficiency of the treated PP filter cloth (**e**) for various oil–water mixtures and (**f**) for the dichloromethane–water mixture after 15 cycles [97].

Similarly, Li et al. [97] achieved superhydrophobic methylated silica with a core–shell structure via a sol–gel process. They initially prepared a silica gel (ca. 110 nm) using tetraethylorthosilicate (TEOS) as a precursor. The superhydrophobic methylated silica sol was then prepared by grafting methyl groups onto the silica gel surface using methyltrimethoxysilane (MTMS), as shown in Figure 15a. The initial silica particles, measuring 100 nm in diameter, exhibited a uniform size distribution and minimal aggregation. Following treatment with MTMS, a thin layer of 5~10 nm formed on the methylated silica, resulting in increased surface roughness compared to the pristine silica, as illustrated in Figure 15b,c. The surface morphology of the methylated silica coating on a PET film, depicted in Figure 15d, revealed a rugged surface with domains composed of numerous small particles. This coating exhibited a highly porous structure with a hierarchical micro/nano design reminiscent of the lotus leaf. The hierarchical pinecone-like structures created numerous grooves, promoting air entrapment and contributing to a more hydrophobic surface. This process enables the direct construction of superhydrophobic and superoleophilic surfaces on various substrates through dip-coating or the doctor-blading method, with a maximum WCA and WSA of 161° and 3°, respectively. The treated substrates demonstrated outstanding water repellence, oil absorption, and oil–water separation efficiency exceeding 96%, as shown in Figure 15e,f.

Liu et al. [98] achieved the synthesis of superhydrophobic graphene aerogel beads (SGA beads) using a one-step, in situ sol–gel method within a coagulation bath containing octadecylamine (ODA). Modification with ODA introduced long alkyl chains with low surface energy onto graphene oxide (GO) sheets, resulting in numerous nanoscale wrinkles on the graphene surface and a remarkable WCA of 153°. The SGA beads exhibited a low density (14.4 mg/cm^3^), high porosity (96.9%), a specific surface area (18.49 m^2^/g), rapid adsorption rates, high adsorption capacity for oils, and recyclability. These characteristics indicate promising applications in the treatment of oils and organic pollutants.

#### 3.2.2. Hydrothermal Technique

**Figure 16:**
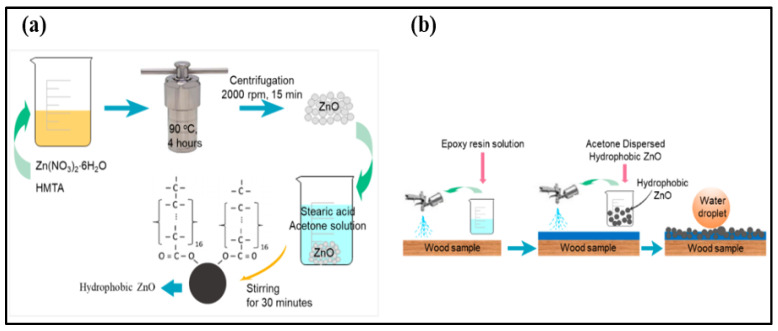
Schematic representation of (**a**) hydrophobic ZnO micro/nanoparticles; (**b**) preparation process of superhydrophobic coatings on wood surfaces [105].

The hydrothermal technique stands as a cost-effective, reproducible, and environmentally friendly approach for synthesizing and modifying materials in an aqueous environment under precisely controlled high-temperature and high-pressure conditions. In this method, precursor materials, frequently metal salts or metal–organic compounds, are dissolved in a solution. Subsequently, they are exposed, alongside a substrate, to elevated temperatures and pressures within a sealed reaction vessel. The rigorous conditions within the hydrothermal environment facilitate chemical reactions and promote the nucleation and growth of materials on the substrate. Hydrothermal techniques are widely employed in various scientific and industrial applications, including the synthesis of nanomaterials, crystal growth, and the modification of surfaces to achieve specific functionalities. In the context of superhydrophobic surfaces, the hydrothermal technique can be utilized to create nanostructures and enhance surface properties, leading to water-repellent characteristics [99,100,101,102,103,104,105,106]. Lan et al. [104] created superhydrophobic surfaces on Al alloy substrates by controlling hydrothermal etching and subsequent 1H,1H,2H,2H-perfluorodecyltriethoxysilane (PFDTES) modification. The resulting surface, featuring hierarchical micro/nanostructures, exhibited remarkable self-cleaning capability, anti-corrosion, antibacterial properties against *E. coli* and *S. aureus* bacteria, and anti-icing properties. Tuong et al. [105] prepared the epoxy@ZnO superhydrophobic coating on *Styrax tonkinensis* wood employing a two-step spray coating method aimed at enhancing wood hydrophobicity and color stability. Initially, hydrophobic ZnO particles in micro/nano sizes were synthesized through a hydrothermal process, followed by stearic acid modification, as shown in Figure 16a. Subsequently, the hydrophobic ZnO particles were applied to incompletely cured epoxy pre-coated wood using a spray gun. The hydrophobic ZnO particles contributed to the formation of multiscale roughness, while the incompletely cured epoxy resin pre-coating enhanced the coating’s durability, as shown in Figure 16b. Notably, the color stability of wood coated with epoxy@ZnO showed a significant improvement, with the total color changes of coated wood samples surpassing those of uncoated wood samples by over approximately 50%. 

Wan et al. [106] crafted a superhydrophobic copper surface for corrosion protection by employing a combination of etching and hydrothermal methods. Initially, copper underwent a 20 h etching process in an ammonia solution, followed by placement in an autoclave with 60 mL of NaOH solution (0.2 mol/L) for a 2 h reaction at 200 °C. The surface modified with stearic acid exhibited superhydrophobicity with a WCA of 157.7 ± 1°. This modified surface demonstrated an impressive corrosion inhibition efficiency of 99.81% in a 3.5 wt% NaCl aqueous solution. Furthermore, the superhydrophobic surface displayed excellent stability in simulated seawater and humid air.

#### 3.2.3. Electrospinning

The electrospinning technique is a versatile method employed to fabricate superhydrophobic surfaces by generating fine fibers through the application of an electric field to a polymer solution or melt. In this process, a syringe containing the polymer solution is subjected to a high voltage, leading to the formation of a charged jet that elongates and eventually solidifies into ultrafine fibers as a result of solvent evaporation or cooling. The resulting fibrous structure, known as a nanofiber mat, creates a high surface area with nanoscale roughness, contributing to the superhydrophobic properties. The morphology of the electrospun fibers is influenced by both the properties of the solution (such as surface tension, viscosity, and conductivity of the polymer solution) and the parameters of the electrospinning process (applied voltage, solution flow rate, and tip-to-collector distance) [107,108,109].

To enhance hydrophobicity, various hydrophobic agents or surface modifiers can be incorporated into the polymer solution. The electrospinning technique allows for precise control over the fiber morphology and surface characteristics, making it a promising method for developing superhydrophobic surfaces with applications in areas such as water repellency, self-cleaning, and oil–water separation [110,111,112,113,114,115,116]. Ding et al. [114] utilized the electrospinning technique to create a superhydrophobic nanofibrous zinc oxide (ZnO) film surface. The process involved electrospinning solutions of poly(vinyl alcohol) and poly(vinyl alcohol)/zinc acetate, followed by a calcination process to produce fibrous zinc oxide superhydrophobic films. By applying a coating of the low-surface-energy material fluoroalkylsilane, the surface wettability of the superhydrophilic ZnO fibrous film (with a WCA~0°) transformed, resulting in a superhydrophobic film (with a WCA~165°) due to the coating of the surface functionalizing agent onto the fibrous films. Similarly, He et al. [115] employed the electrospinning method to prepare a polyvinylidene fluoride (PVDF) nanofibrous membrane featuring superhydrophobic/superoleophilic properties for efficient oil–water separation, coupled with antibacterial capabilities. Modification of the electrospun PVDF nanofibrous surface was achieved through the incorporation of ZnO nanoparticles, tannic acid (TA), and n-dodecyl mercaptan (DT), aimed at reducing surface energy and enhancing surface roughness. The resulting nanofibrous membranes exhibited a water contact angle of 156.5° and a tensile strength of up to 69.25 MPa. The mechanically robust superhydrophobic nanofibrous membrane demonstrated an oil–water separation efficiency exceeding 99%, accompanied by a notable flow flux of 1008.88 L·m^−2^ h^−1^. Additionally, antimicrobial testing revealed a bacteriostatic rate surpassing 98%.

**Figure 17:**
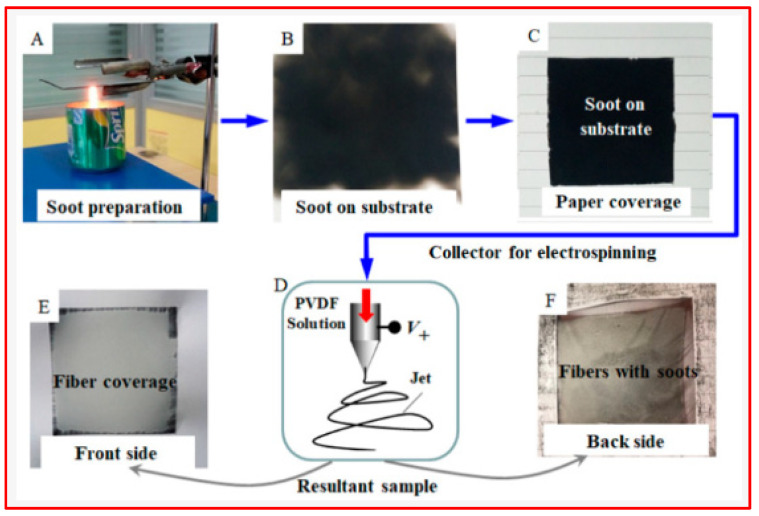
Process for preparing candle soot and superhydrophobic poly(vinylidene fluoride) (PVDF) membranes: (**A**,**B**) collection of candle soot on sheet metal over the flame; (**C**) paper adhered to the sheet metal; and (**D**) schematic of the electrospinning process using paper and metal as substrates with the deposited fibrous membrane. The soot is pasted to the back side (**F**) of the membrane rather than the front side (**E**) [116].

Cai et al. [116] innovatively employed a technique inspired by the traditional Chinese hand-stretched noodle-making process to create a superhydrophobic polyvinylidene fluoride (PVDF) membrane. In this novel method, nanofibers were electrospun onto a substrate covered with (super)hydrophobic nanopowders (candle soot), as shown in Figure 17. 

This approach enhanced the superhydrophobic properties of the electrospun nanofibrous membranes as some nanopowders adhered to the fiber surface. Additionally, heating the substrate enhanced the bonding of nanopowders, allowing them to potentially become embedded within the nanofibers. This advancement in the electrospinning process holds significant promise for various membrane applications.

#### 3.2.4. Chemical Vapor Deposition (CVD)

Chemical vapor deposition (CVD) stands as a vacuum deposition technique based on the chemical reactions of gaseous precursors on a substrate surface. In this method, precursor gases containing the desired elements react on the substrate surface, resulting in the formation of a thin and uniform layer of the intended material. The initiation of the reaction involves heating the substrate, with the chemical reactions unfolding in the gas phase. The volatile by-products are subsequently eliminated, leaving the deposited material in place. 

While CVD provides meticulous control over film characteristics such as thickness, composition, and crystal structure, it is a time-consuming and costly process. A notable challenge with CVD lies in its limitation for obtaining large-sized samples. Despite these drawbacks, the versatility of CVD makes it a preferred method for depositing diverse materials, including superhydrophobic coatings [117,118,119,120,121,122,123,124]. This technique proves effective in generating rough surfaces by organizing micro-/nanoparticles into ordered structures or depositing a thin layer of low surface energy materials onto a pre-existing rough surface. 

**Figure 18:**
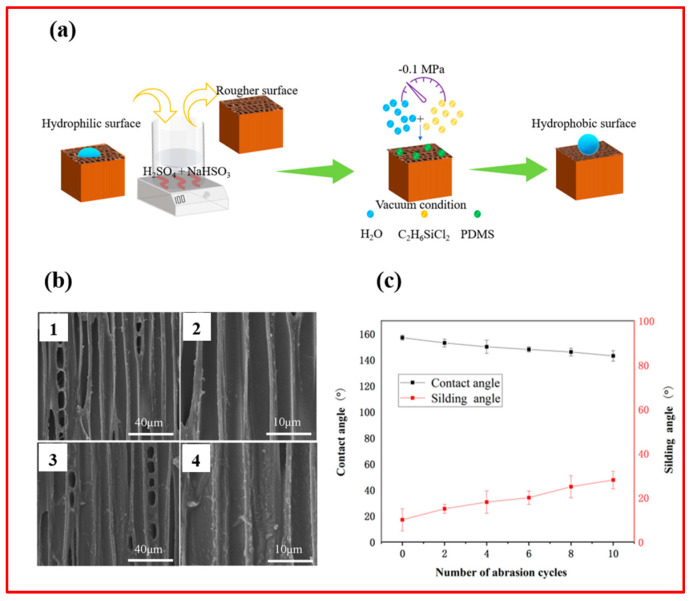
(**a**) Schematic representation outlining the complete process for fabricating a PDMS@wood coating; (**b**) SEM images of wood samples on tangential section: (1,2) untreated wood; (3,4) PDMS@wood; and (**c**) WCA and SA variations with the number of abrasion cycles for the hydrophobic wood surface [123].

Cai et al. [122] demonstrated the fabrication of transparent superhydrophobic hollow films by using candle soot as a template, followed by CVD of methyltrimethoxysilane (MTMS) and calcination at 450 °C. They optimized the deposition time concerning the transparency and superhydrophobicity of the films. The resulting superhydrophobic films, when coated on a glass substrate exhibited a high transmittance of approximately 90%, with a WCA of more than 165.7° and a SA of about 2.1°. Furthermore, these films displayed remarkable thermal stability and effective moisture resistance even after calcination at temperatures as high as 500 °C. Yang et al. [123] introduced an economical approach to enhance the hydrophobicity of wood using a simple low-temperature CVD technique. The process involved using dichlorodimethylsilane as the CVD chemical source to coat the wood with polydimethylsiloxane (PDMS@wood), resulting in a hydrophobic surface, as depicted in Figure 18a. SEM images reveal that on the tangential section, the wood vessel wall of untreated wood was smooth and distinct (Figure 18b(1,2)). The PDMS@wood cell wall exhibited a particulate morphology on the surface (Figure 18b(3,4)), providing high roughness crucial for forming the hydrophobic surface. The granular and particulate materials were silicon from PDMS, confirmed by EDS analysis of the PDMS@wood samples. The prepared PDMS@wood demonstrated a WCA of 157.28° along with notable thermal stability. Significantly, even after extended storage (30 days) and subjected to a sandpaper abrasion test (Figure 18c), PDMS@wood retained its excellent hydrophobic properties, indicating considerable potential for large-scale industrial production.

Recently, Huang et al. [124] introduced a facile one-step method for fabricating transparent superhydrophobic coatings with customized nanocone array structures using initiated chemical vapor deposition (iCVD). This innovative iCVD process employs condensed nanosized monomer droplets as nucleation centers, leading to the vertical growth of polymer nanocones through a proposed “vapor–liquid-solid” mechanism. The deposition conditions can be tailored to control both the height and density of the nanocones. The optimized nanocone array coating exhibits outstanding water repellency, along with superior anti-icing and anti-frosting capabilities, all while maintaining high light transmittance.

#### 3.2.5. Electrochemical Deposition 

Electrochemical deposition serves as a versatile and controlled approach for creating superhydrophobic surfaces by electrodepositing materials onto conductive substrates. In this method, the substrate acts as the cathode, and the material to be deposited dissolves in an electrolyte solution. Upon applying an electric current, metal ions from the solution are reduced at the cathode, resulting in the formation of a solid layer on the substrate. Precise adjustment of deposition parameters, such as current density, deposition time, and bath composition, enables the creation of hierarchical micro/nanostructures contributing to surface superhydrophobicity. The technique is not only fast, reproducible, and scalable but also allows the fabrication of diverse surface morphologies like rods, sheets, tubes, fibers, cones, and needles by modifying deposition conditions [125]. Superhydrophobic surfaces produced through electrochemical deposition possess potential advantages, offering ease of large-scale morphology creation and ensuring strong bonding between materials, resulting in durable coatings.

This method provides precise control over surface morphology, composition, and roughness, offering flexibility in tailoring superhydrophobic coatings for applications such as self-cleaning, anti-fouling, and water-resistant materials [126,127,128,129,130,131,132]. The electrochemical deposition technique stands as an advantageous avenue for developing customized superhydrophobic surfaces with enhanced functionalities. Li et al. [131] successfully generated a superhydrophobic film with hierarchical porous structures using Fe–myristic acid on a copper substrate through a straightforward one-step electrochemical deposition process. In this electrodeposition procedure, two copper plates served as the cathode and anode, with the electrolyte being an ethanol solution containing Fe–myristic acid. The resulting coating, obtained with an electrodeposition time of 10 min at 20 V, exhibited a maximum contact angle of 159.2° and a minimum sliding angle of 1.7°. Even after undergoing 105 cm abrasion tests, the contact angle of the superhydrophobic film remained at 153.17°, showcasing robust mechanical properties. The superhydrophobic matrix significantly enhanced the corrosion resistance of copper in various environments, including atmospheric conditions, natural seawater, and salt spray. This highlights that the superhydrophobic matrix provides substantial abiotic corrosion inhibition to the underlying copper metal, with an impressive inhibition efficiency of 99.85% observed after 28 days of immersion in seawater. 

**Figure 19:**
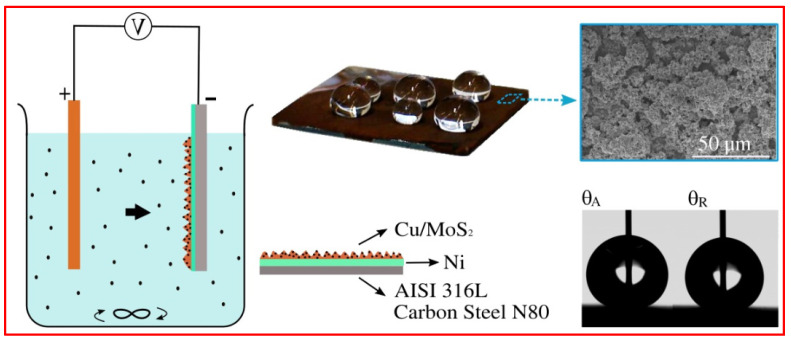
The preparation process of the superhydrophobic Cu–MoS_2_ composite coating on the substrate [132].

Prado et al. [132] developed a superhydrophobic coating using composite electrodeposition, incorporating MoS_2_ particles into a copper matrix. AISI 316L stainless steel and N80 carbon steel were chosen as substrates, with a thin electrodeposited Ni layer to improve coating adhesion. The resulting coating exhibited a lotus hierarchical coral-like structure, composed of composite Cu and MoS_2_ protuberances. The electrodeposition process enabled control over surface roughness and surface energy, with the latter dependent on the quantity of MoS_2_ particles in the coating. The Cu–MoS_2_ composite coating achieved advancing contact angle values of up to 158.2° with a contact angle hysteresis of 1.8°, as depicted in Figure 19.

#### 3.2.6. Layer-by-Layer Deposition

Layer-by-layer (LbL) deposition is a simple, low-cost, and substrate-independent technique employed in the fabrication of superhydrophobic surfaces by sequentially adsorbing layers of oppositely charged materials onto a substrate. In this process, the substrate is alternately dipped into solutions containing positively and negatively charged species, creating a multilayered thin film. The coated film is then rinsed in water and dried after each dip [133]. These films can consist of various materials, such as polymers or nanoparticles, depending on the desired properties of the superhydrophobic surface. The layering process allows precise control over the thickness, composition, and surface morphology of the deposited films. By carefully selecting materials with appropriate surface energies and roughness, the resulting surface can exhibit exceptional water repellency with high contact angles and low sliding angles. 

The layer-by-layer deposition technique provides a flexible and scalable approach for tailoring superhydrophobic surfaces, making it applicable in various industries, including self-cleaning, anti-fouling, and water-resistant coatings [134,135,136,137,138,139,140]. However, the major disadvantage of this technique is that it is time-consuming and applicable to limited materials. Shao et al. [139] utilized a layer-by-layer assembly method to create a superhydrophobic coating on a wood surface. The process involved the sequential auto-deposition of polydopamine (PDA) and 1H,1H,2H,2H-perfluoro-decyl trichlorosilane (PFDTS). Initially, PDA was deposited on the wood surface, forming a highly cross-linked granular structure that enhanced the surface roughness of the wood. Subsequently, the wood surface underwent further modification with the low-surface-energy PFDTS, resulting in a superhydrophobic wood surface with a water contact angle (WCA) of 154° after the PDA deposition. The coating introduced protrusions and microgrooves through cell walls and cavities in the wood, avoiding the use of solid particles for surface roughening. The deposition of PDA on the wood surface led to the formation of natural micro/nanoparticles with a uniform distribution. 

Syed et al. [140] introduced a layer-by-layer (LbL) spin-assembled coating composed of polyaniline–silica composite and tetramethylsilane functionalized silica nanoparticles (PSC/TMS-SiO_2_) to achieve a synergistic effect of superhydrophobicity and enhanced anti-corrosion properties on stainless steel surfaces. Notably, the hierarchical integration of these two coating materials with distinct surface roughness and energy in a multilayer structure allowed the wetting feature to transition from a hydrophobic to a hydrophilic state by modulating the layer count (n) with decreasing hydrophilicity. Surfaces with an odd n (TMS–SiO_2_ surface) exhibited hydrophobic characteristics, while those with an even n (PSC surface) displayed hydrophilic traits. The coating demonstrated superhydrophobicity and self-cleaning abilities with a high water contact angle (CA) of 153° ± 2° and a minimal sliding angle (SA) of 6° ± 2°, attributed to the synergy of surface composition and roughness. Beyond its self-cleaning behavior, the coating exhibited significantly enhanced corrosion resistance against aggressive media, maintaining stability even after 240 h of exposure, attributed to superhydrophobicity and an anodic shift in corrosion potential. Table 3 shows the advantages and disadvantages of the superhydrophobic preparation process during practical production.

## 4. Applications

**Figure 20:**
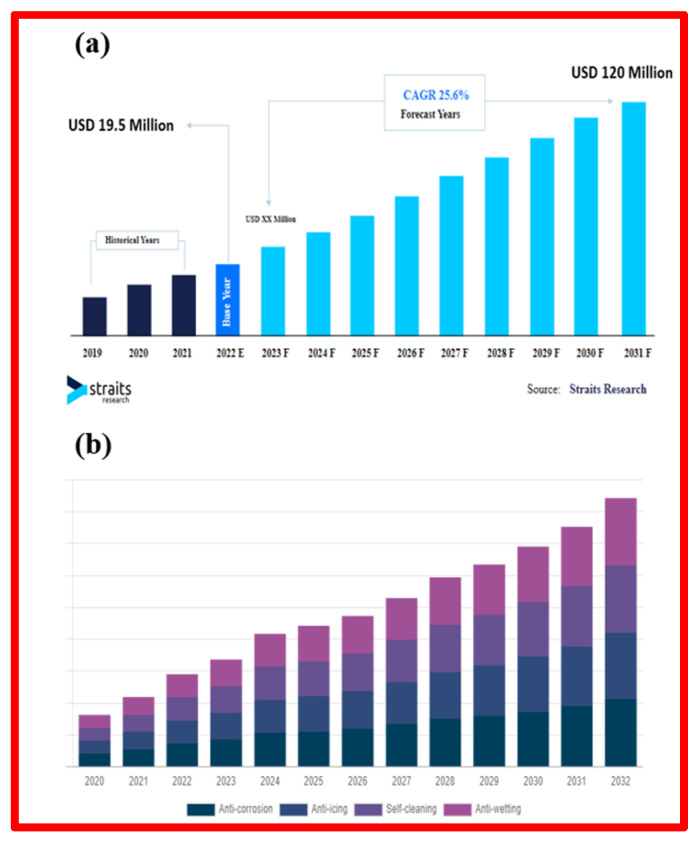
Commercialization status of superhydrophobic coatings: (**a**) global market value of superhydrophobic surfaces; (**b**) by property.

Superhydrophobic surfaces have made great progress in the past two decades. The global superhydrophobic coating market has been experiencing significant growth due to the increasing demand across various industries and is expected to witness tremendous revenue opportunities in the upcoming years. These coatings find applications in sectors like automotive, aerospace, electronics, textiles, and construction, among others. As of the most recent market analysis published by Straits Research, the global superhydrophobic coatings market was valued at approximately USD 19.5 million in 2021 and is expected to reach around USD 120 million in 2030, at a compound annual growth rate (CAGR) of 25.6% between 2022 and 2030, as shown in Figure 20a [141]. 

Superhydrophobic coatings are formulated to repel water and various liquids, providing attributes like self-cleaning, anti-corrosion, and anti-icing functionalities. In terms of product classification, the global market for superhydrophobic coatings is categorized into anti-corrosion, anti-icing, self-cleaning, and anti-wetting. The segment related to anti-wetting demonstrates the most significant market share and is projected to experience a CAGR of 26.1% throughout the forecast period, as illustrated in Figure 20b [141].

**Figure 21:**
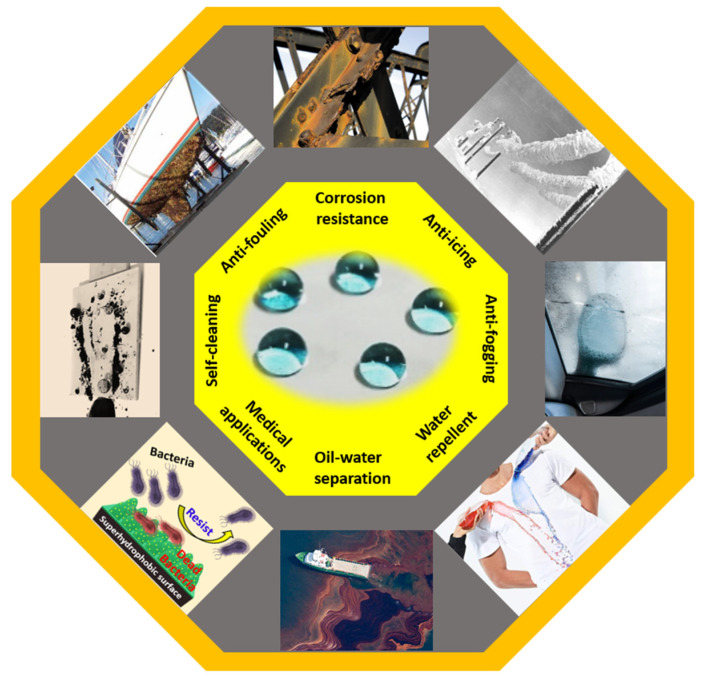
Nature-inspired artificial superhydrophobic surfaces with diverse applications.

Much research has been carried out to discover methods for developing superhydrophobic surfaces that can be used in a variety of industries for commercialization [142,143,144,145,146]. Therefore, we primarily discuss the uses of superhydrophobic coatings for self-cleaning, anti-icing, anti-fouling, oil–water separation, anti-fogging, anti-corrosion, the medical industry, and other domains, as shown in Figure 21.

### 4.1. Self-Cleaning 

Self-cleaning materials are designed to be effortlessly cleansed with water, which offers diverse applications while reducing maintenance costs and having an environmental impact. As a result, self-cleaning surfaces are being extensively researched and commercialized for use in solar energy panels, paints, windows, satellite dishes, and windscreens for automobiles [108,147,148,149,150,151]. Textile materials that include self-cleaning properties become wash-free or require fewer washings, which makes them convenient for daily usage and adds value to the product. This phenomenon is known as the lotus effect, which was first identified by Neinhaus and Barthlott on the surface of lotus leaves and was later patented in 1998.

**Figure 22:**
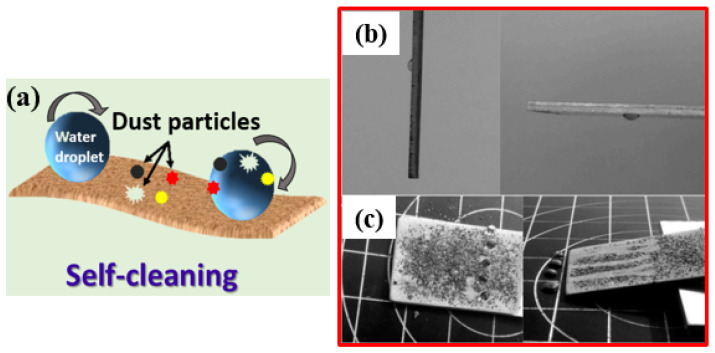
(**a**) Diagram illustrating the self-cleaning functionality of artificial superhydrophobic surfaces. A water droplet, featuring a notably high water contact angle (WCA), effortlessly rolls across the surface, effectively carrying away any adhered dirt or contaminants present on the superhydrophobic surface. The self-cleaning capability of aluminum alloy samples with (**b**) original sample (OS) and (**c**) binary structure (BS) obtained via a multi-step modification process [156].

Superhydrophobic surfaces exhibit a distinctive set of characteristics, including high water contact angles (WCA > 150°) and low roll-off angles, facilitating the efficient self-cleaning mechanism, as shown in Figure 22a [151,152]. This self-cleaning ability is attributed to the surface’s microstructures and nanostructures, which form a unique morphology. Upon contact with water droplets, the surface induces the formation of nearly spherical shapes, minimizing the contact area and enabling easy rolling off of the droplets. This process effectively removes dust, dirt, and contaminants from the surface. The microscale and nanoscale features not only contribute to reduced water adhesion but also enhance the self-cleaning effect. The Cassie–Baxter wetting state, characterized by trapped air pockets between the water droplet and the surface, further aids in minimizing contact and adhesion, solidifying the surface’s impressive self-cleaning capabilities. Additionally, these surfaces exhibit stain-resistant properties due to their small contact areas and high contact angles [153].

Wang et al. [154] developed A-SiO_2_/N-TiO_2_@HDTMS coatings onto the substrate surface with a water contact angle (WCA) of 157.2° and contact angle hysteresis (CAH) of 2.7°, demonstrating prolonged outdoor self-cleaning functionality. Similarly, Xu et al. [155] employed a sustainable approach using plant-derived phytic acid to etch cotton fabric to enhance its surface roughness. Coated with thermosets derived from epoxidized soybean oil and then covered with stearic acid (STA), the modified fabric achieved a water contact angle of 156.3° and exhibited exceptional superhydrophobic qualities against liquid pollutants and solid dust.

Shao et al. [157] investigated a novel water-based superhydrophobic coating using polytetrafluoroethylene (PTFE) doped with graphene. The study involved mixing PTFE with graphene nanoparticles to create a PTFE–graphene composite, which was uniformly dispersed in aqueous fluorocarbon resin (FEM-101A-2-SFT) and applied to the substrate. The results revealed that graphene was evenly distributed on the PTFE surface, and the graphene–PTFE composite was immobilized on the coating surface through self-cross-linking fluorocarbon resin. This process generated numerous nanopulled structures on the coating surface, achieving a water contact angle of 153°. The resulting superhydrophobic coating exhibited excellent adhesion and corrosion resistance properties. After 40 cycles of mechanical wear and 4 h of water impact, the water static contact angle remained at 120° and 110°, respectively. The coating demonstrated 5B-level adhesive force according to ASTM standards and exhibited outstanding self-cleaning abilities. The self-cleaning effect was evident when dust was sprinkled on the surface, as water droplets rolled off easily, carrying away the dust and leaving the coating surface clean. This self-cleaning property is attributed to the nanoprotruding structure and low surface energy of the coating surface, preventing water permeation.

Similarly, the self-cleaning performance of aluminum alloy samples was investigated, revealing enhanced capabilities with micro/nanoscale binary structures [154]. As shown in Figure 22b, a water droplet was sprayed on the surface of the untreated sample. The water droplet could not slide down, not even if the sample was tilted to 90° or 180°. Whereas, water droplets on the sample with micro/nanoscale binary structures (BS) were found to slide down smoothly when the sample was tilted at an angle of 2.5° ± 0.7° and carried away brown alumina powders with them (Figure 22c). Table 4 summarizes past research on self-cleaning applications [158,159,160,161,162].

### 4.2. Anti-Icing 

The issue of icing on various surfaces poses significant challenges in several technological domains, leading to problems such as increased weight affecting electrical equipment, wind turbine blades, and airplane wings [163,164]. Even on hot days, air conditioners and refrigerators may encounter reduced efficiency when cooling and evaporation coils ice up. The electrothermal method has proven effective in accelerating ice melting. While salt is commonly used to melt snow and ice on roads during the winter, its high solubility poses environmental concerns, as approximately 90% of the salt may runoff, causing dehydration to aquatic life and vegetation along roadsides.

Physical mechanical deicing is the easiest method, but it is limited to easily accessible equipment. This approach becomes challenging when the ice-covered surface is not easily reachable [35]. Moreover, mechanical deicing is considered unsafe and unreliable due to the risk of damaging equipment during the deicing process. In recent times, researchers have directed their efforts towards creating materials characterized by low ice-adhesion strength. This property allows ice formed on these materials to be effortlessly removed either by its own weight or through the action of natural wind.

**Figure 23:**
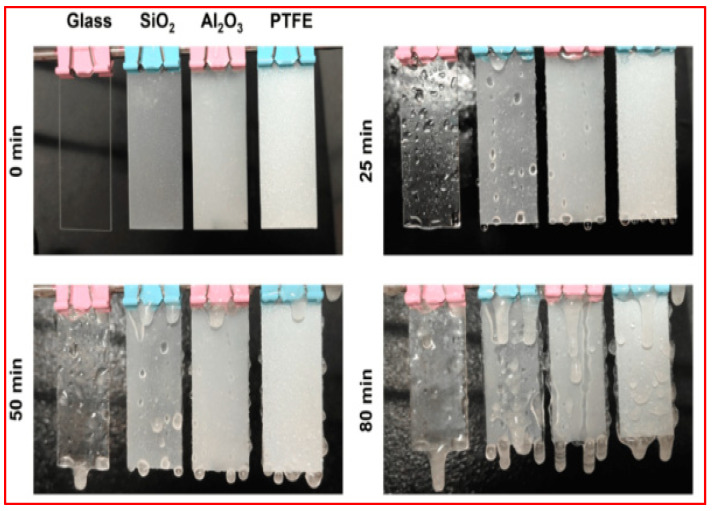
The glaze icing process on bare glass and three different coatings on a glass surface [167].

Zheng et al. [165] presented a method for surface processing titanium alloy using femtosecond laser technology, followed by dual-layer coating functionalization to create superhydrophobic surfaces resembling lotus leaves with enhanced anti-icing properties. Femtosecond laser treatment produced periodic microstructures on the titanium alloy surface. Subsequently, a dual-layer coating was sprayed on the surface, creating a low-surface-energy layer and nanostructure. The formed surface exhibited excellent superhydrophobicity, with an enhanced contact angle of 165° and a reduced sliding angle of 1.2°. Xiao et al. [166] propose a quick and easy method for creating anti-icing/deicing coatings using a two-step deposition process involving a Fe_3_O_4_@PDMS mixed liquor. This approach quickly produced a photothermal superhydrophobic coating, leveraging the excellent photothermal conversion properties of Fe_3_O_4_ particles to impart a micro/nanoscale rough structure to the coating surface. PDMS is used as a binder to increase the coating’s durability while giving it low surface energy. Experimental results demonstrate that when exposed to simulated sunshine for 10 min, the surface temperature of the coating can reach 85 °C, effectively melt the frozen droplets within 270 s. With a water contact angle of 160°, the resulting coating exhibits outstanding superhydrophobicity and can delay icing time by up to 660 s. Whereas, by employing a one-step spraying method, superhydrophobic coatings composed of PTFE, Al_2_O_3_, and SiO_2_ were successfully applied to glass slides [167]. Evaluation of experiments carried out in an artificial climate chamber (Figure 23) revealed that these three superhydrophobic coatings outperformed glass in resisting glaze icing. This enhanced performance is attributed to their exceptional superhydrophobicity, which facilitates the easy rolling away of water droplets from the surface, significantly reducing contact duration and minimizing the likelihood of freezing. Table 5 provides an overview of past research on anti-icing applications [168,169,170,171,172].

Wang et al. [173] introduced a two-step spraying process for the preparation of plasmonic photothermal superhydrophobic coatings, incorporating MXene@Au hybrids with waterborne polyurethane. MXene (Ti3C2Tx) exhibits a localized surface plasmon (LSPR) enhancement effect and a broad absorption band [174,175]. Additionally, its good electrical conductivity allows applications in flexible electronic devices and soft robots [176]. Due to its exceptional optical properties and rapid heat transfer capabilities, MXene finds extensive use in light absorption and light-to-heat conversion devices [177,178]. To attain superhydrophobicity, chemically modified SiO_2_ nanoparticles were applied to the MXene@Au-WPU layer, resulting in a fSiO_2_/MXene@Au-WPU (fluoroalkyl silanes-SiO_2_/MXene@Au-WPU) superhydrophobic photothermal coating with a contact angle of 153°. The composite coating proved effective for anti-icing and deicing applications, displaying an ultra-long anti-icing duration of 1053 s under low-temperature and high-humidity conditions (−20 °C, relative humidity 68%). Compared to previous studies, the coating also demonstrated an exceptionally high photothermal deicing efficiency of 73.1%. The coating’s high photothermal properties facilitate rapid ice melting in the irradiated area, aided by the superhydrophobic characteristics that guide melted water to slide off. Additionally, the results show that the coating’s resistance to corrosive liquids is within a pH range of 1 to 13.

### 4.3. Anti-Fogging 

Fogging is the term used to describe the phenomenon wherein the temperature differential between the surface and the humid environment causes humid air to condense as discrete, small drops of water on optical surfaces. The dispersion of incident light by these water droplets restricts its transmission or reflection on solid surfaces, leading to blurred vision. Fog can accumulate on optical surfaces, including camera lenses, binoculars, swimming goggles, eyeglass lenses, and bathroom mirrors. Variables such as temperature, humidity, and airflow influence the occurrence of fogging. In addition to being annoying, fogging may cause additional problems in a variety of applications, including safety. Fogging, for example, might impair vision during endoscopic surgery and raise the possibility of a failure of the operation [179]. The formation of fog on moving car windscreens and motorcycle helmet visors is intimately associated with road safety. Additionally, fogging on greenhouse cladding materials reduces the amount of light that reaches the crops, which has an impact on agricultural productivity. Furthermore, fogging lowers solar panel efficiency. For various optical applications, it became highly desirable to eliminate or reduce the fogging phenomenon. To mitigate or eliminate fogging in optical applications, researchers commonly employ cost-effective and durable techniques such as applying coating layers to surfaces or modifying them chemically or physically [180,181].

**Figure 24:**
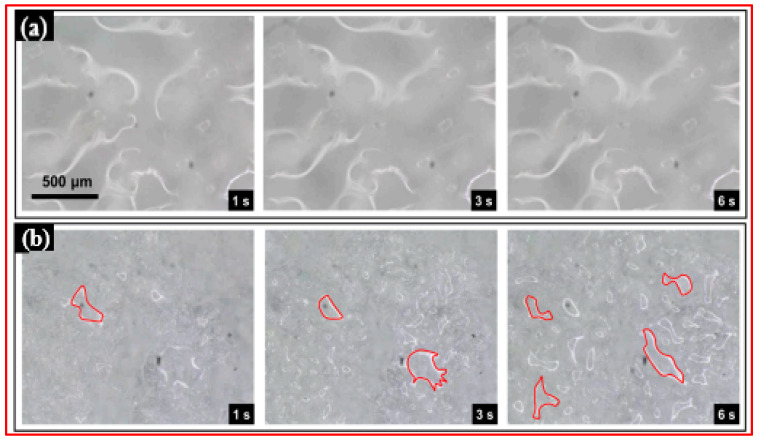
Anti-fog test on the surface of a (**a**) flat plate and (**b**) BFRSs [184].

Huang et al. [182] reported the use of diamond as an optical coating for challenging applications, like air force optical windows and offshore oil exploitation. After being treated with oxygen plasma, the diamond films became superhydrophilic, which caused the contact angle to decrease from 87° to less than 5° and resulted in antifogging activity. On the other hand, an ultrathin diamond coating on a quartz slide showed an oil contact angle and a di-chloromethane droplet contact angle of 153° and 157°, respectively, on the O_2_-plasma-treated surface. Steaming and freezing tests demonstrated that the coated samples maintained sufficient transparency and anti-fogging activity. Notably, the uncoated sample took 3.5 min to evaporate, while the fog on the diamond thin film evaporated in just 4 s. Varshney and Mohapatra [183] discussed the fabrication of superhydrophobic coatings on brass surfaces using both two-step (chemical etching with a hydrochloric and nitric acid mixture, following a treatment with lauric acid) and one-step (treatment with lauric acid) methods. Treated brass surfaces exhibited rough microstructures, resulting in superhydrophobicity with water contact angles exceeding 173° and sliding angles below 4°. In addition to that, the formed coatings demonstrated anti-fogging and self-cleaning properties. In another approach, combined sol–gel and biotemplating techniques were used to fabricate bio-inspired anti-fogging and anti-reflection surfaces (BFRSs) with multiscale hierarchical columnar structures (MHCS) [184]. The formed surface exhibited rapid elimination of dispersed fog droplets within 6 s (Figure 24). The effective anti-fogging performance of the surface was attributed to the capillary force imbalance of the MHCS acting on the liquid film.

### 4.4. Oil–Water Separation 

The rapid pace of industrialization, global population growth, and the expansion of the worldwide economy have resulted in the contamination of water, which has emerged as a significant and escalating environmental issue. Oily wastewater is a critical form of water pollution and is commonly produced by industries, oil spills, automotive transportation, and domestic sewage [185,186]. The excess presence of oils poses a severe threat to the environment, which adversely affects aquatic life such as fish, animals, and birds, making them more susceptible to hypothermia and negatively impacting plant growth. Recently, on 25 July 2020, the Japanese-operated MV Wakashio bulk carrier ship collided with a coral reef on the island of Mauritius, causing severe damage to the rich marine ecosystem. 

Various methods were reported for the removal of oil and grease from wastewater, including dispersion [187,188], adsorption [189,190], flotation [191,192], biological treatment [193,194], and burning [195]. However, these approaches may come with drawbacks such as low stability, inefficiency, high energy consumption, expensive chemicals, and the potential for secondary pollution. Desirable oil–water emulsion separation technologies should possess characteristics such as excellent stability, cost-effectiveness, easy operation, high separation efficiency, substantial permeation, and minimal secondary contamination throughout the synthesis and separation processes [196]. 

**Figure 25:**
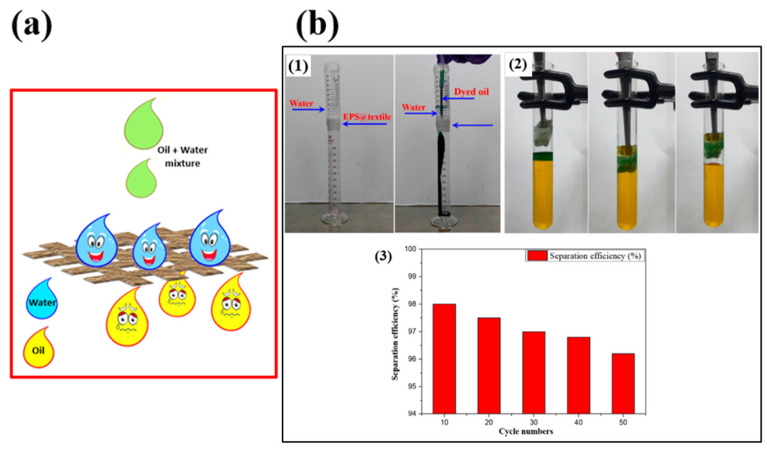
(**a**) Oil–water separation mechanism of superhydrophobic surfaces; (**b**) picture of oil–water separation using an EPS@textile-coated fabric as a (1) filtering membrane; (2) adsorbent; and (3) separation efficiency of the coated fabric [200].

In response to such challenges, various functional materials with superhydrophobic and superoleophilic properties have undergone extensive study for efficient oil–water separation filtration. These materials include meshes, membranes, sponges, foams, and fabrics. The operational principle of superhydrophobic surfaces in oil–water separation encompasses the synergistic effects of superhydrophobicity, oleophilicity, and distinctive surface structures. This combination enables the selective repulsion of water, the attraction and retention of oil, and the facilitation of a self-cleaning mechanism, ensuring a highly effective and environmentally sustainable separation process, as shown in Figure 25a. Cai et al. [197] utilized natural balsa to create a highly flexible and durable superhydrophobic polydivinylbenzene (PDVB)-wood membrane. The membrane features hydrophobic nanopores achieved by coating porous wood with cross-linked PDVB. In air, the PDVB-wood membrane exhibits unique oil wettability with a 0° oil contact angle. The membrane also demonstrates exceptional WCA exceeding 160° and a low WSA of 3.5°. Remarkably, water droplets exhibit bouncing behavior when quickly dropped onto the PDVB–wood membrane. The superhydrophobic properties endure rigorous mechanical and chemical tests, including sandpaper rubbings, tape sticking, and exposure to acid, alkali, and salt solutions, highlighting their durability and stability. With a separation efficiency exceeding 99.98% for surfactant-stabilized water-in-oil emulsions and a high flux of up to 8829.4 L m^−2^ h^−1^bar^−1^, the membrane maintains high efficiency even after 20 separation cycles. The remarkable separation performance of the PDVB-wood membrane is attributed to the synergistic effects of superhydrophobicity and nanopores. By combining superhydrophobicity/superoleophilicity with nanoscale pores, the membrane prevents micro/nano-scale water droplets in water-in-oil emulsions from penetrating while allowing free penetration of oil due to its superoleophilicity. This achievement fulfills the demulsification and separation objectives.

Jie et al. [198] used an electrodeposition approach to prepare micro/nano superhydrophobic structures on a copper mesh surface, employing choline chloride/ethylene glycol ionic liquid as an electrolyte. The contact angle of the electrodeposited copper mesh reached 152°, showing superior performance compared to pure copper mesh. Demonstrating a separation efficiency exceeding 95%, the superhydrophobic copper mesh exhibited exceptional oil–water separation capabilities across various oil types. In another study, a simple spraying method was used to prepare a superhydrophobic TiO_2_/SiO_2_ nanoparticle coating on sponge, loofah, and metal mesh surfaces. The coating exhibited a surface with convex nano-nipple and nano-scale pore structures, providing microstructural evidence of its superhydrophobic properties, with a water contact angle (WCA) of ≥155° and an oil contact angle (OCA) close to 0°. Experimental investigations involving different oil–water mixtures revealed an oil–water separation effectiveness of approximately 95% for the superhydrophobic coating on various substrates. Remarkably, even after 60 separation cycles, the coating maintained a separation efficiency above 90%, indicating its enhanced durability [199]. Kao et al. [200] used a simple painting process to produce a new micro/nanostructure by coating ethylenediaminetetraacetic acid/poly(dimethylsiloxane)/fluorinated SiO_2_ (EDTA/PDMS/F-SiO_2_) on a textile surface. The result shows the high oil–water separation efficiency of the superhydrophobic EPS@textile, even in the absence of external force (Figure 25b). Table 6 summarizes past research on oil–water separation applications [158,160,201,202,203].

### 4.5. Anti-Fouling

The accumulation of undesired substances on different surfaces submerged in water is known as fouling. Both inorganic and organic substances, as well as living organisms (biofouling), can be considered fouling materials. Biofouling is a major problem that causes a lot of problems in the maritime environment. Seawater can become contaminated by coatings formed by non-target species and fouling, which provide severe toxicity concerns for marine life. It is now necessary to develop environmentally friendly anti-fouling solutions due to the increasing significance of environmental protection. Researchers are actively exploring biomimetic antifouling coatings, which mimic the micro-structured surfaces found in marine life, as a way to solve the environmental issues related to traditional coatings [204]. Microbial biofouling poses a significant threat to numerous other environmentally related systems, such as water treatment and distribution systems, heat exchangers, and many more. These systems are susceptible to several issues caused by biofouling, including reduced heat transfer efficiency, blockages in pipes, power failures, high maintenance costs, and serious mishaps. As a result, it is preferable to create surfaces with anti-fouling properties using an easy and affordable technique.

Yin et al. [205] introduced an economical and fluorine-free technique for coating superhydrophobic Ni_3_S_2_ on 304 stainless steel. The resulting superhydrophobic coating significantly impeded water intrusion and exhibited remarkable resilience in maintaining its superhydrophobicity even after exposure to conditions such as heating up to 300 °C or prolonged submersion in ethanol and undergoing five cycles of O_2_ plasma etching with heating treatment. Anti-fouling tests validated the effectiveness of the superhydrophobic coating in forming a protective barrier against contamination on the steel surface. In a separate study, Shi et al. [206] developed a superhydrophobic and antibacterial membrane using underwater adhesion technology for directly immobilizing it on substrates in seawater. The membranes displayed excellent hydrophobicity, mechanical strength, and flexibility, boasting a water contact angle (WCA) of 161.3° and a sliding angle (SA) of 4.9°. These qualities remained stable even after 30 days of submersion in seawater, affirming their prolonged service life. 

**Figure 26:**
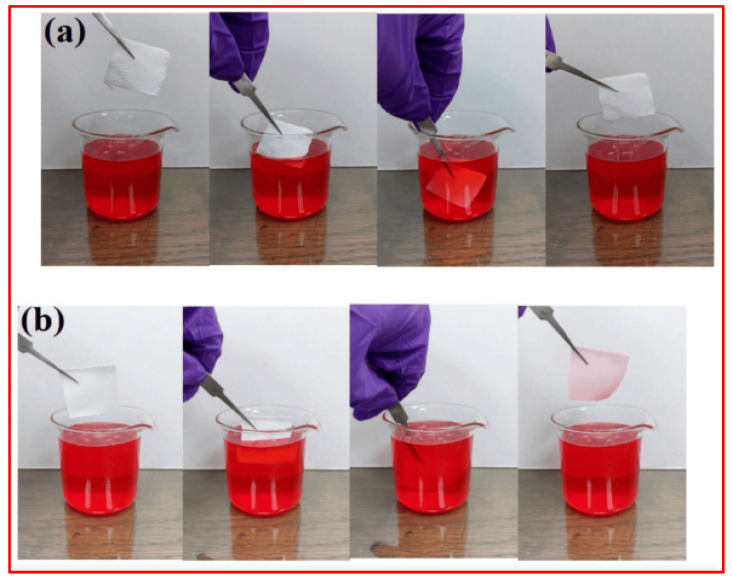
Picture showing the antifouling performance of (**a**) EPS@textile and (**b**) bare textile against water [200].

This new approach has general relevance in ocean engineering and may be applied to different substrates to generate antifouling coatings in seawater. Furthermore, research was conducted on the coated fabric surface’s ability to inhibit the growth of rhodamine B liquid pollutants [200]. Figure 26a illustrates that the superhydrophobic textile remained remarkably clean, contrasting with the evident pollution of the non-superhydrophobic textile, as shown in Figure 26b. This finding shows the outstanding antifouling property of fabricated superhydrophobic EPS@textile. The unique combination of superhydrophobic characteristics and high hygroscopicity of the uncoated fabric prevented the colored water from permeating the coated superhydrophobic fabric by trapping air.

### 4.6. Anti-Corrosion 

**Figure 27:**
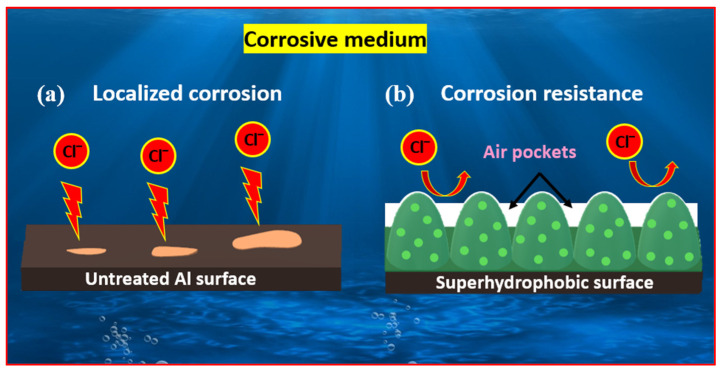
The anti−corrosion mechanism of the superhydrophobic surfaces.

Corrosion refers to the deterioration of a metal surface due to chemical or electrochemical reactions with the environment. The practical utility of metals and metal alloys is constrained by their susceptibility to corrosion, resulting in substantial financial losses and environmental pollution. Consequently, extensive research efforts, supported by significant funding, are underway to develop anti-corrosive materials. Although chromium-containing compounds are conventionally used for corrosion prevention, their negative impact on both human health and the environment prompts the exploration of alternative methods. One such approach involves directly creating superhydrophobic coatings on metal surfaces to enhance their anti-corrosion properties [207]. This approach utilizes the creation of an air layer between the structured superhydrophobic surface and the solution. These trapped air pockets act as a protective barrier, effectively preventing the corrosive medium from reaching the surface, as depicted in Figure 27.

Superhydrophobic coatings have become widely used to enhance the corrosion resistance of various surfaces, including steel, alloys (Al, Cu, Zn, and Fe), and titanium (Ti). Huang et al. [208] introduced an environmentally friendly surfactant-free nanoprecipitation method to fabricate a nanocomposite coating based on polyaniline-titanium oxide (PANI-TiO_2_). Incorporating two sizes of TiO_2_ nanoparticles enhanced the coating’s hydrophobicity and conductivity. Compared to traditional epoxy coatings, the resulting coating with hierarchical micro/nanostructures demonstrated higher water contact angles (>150°) and superior anti-corrosion properties. 

Microplastics are commonly found in saline water, emphasizing the need to study the anticorrosion properties of surfaces in such environments. Rius-Ayra et al. [209] introduced a robust, non-fluorinated superhydrophobic surface through an anodizing process and liquid-phase deposition (LPD) of lauric acid for surface functionalization and superwettable properties. This combined approach resulted in outstanding anticorrosion performance of the metallic substrate in NaCl aqueous solution. The hierarchically structured superhydrophobic surface achieved a water contact angle (WCA) of 154°, a sliding angle (SA) of 1°, and a contact angle hysteresis (CAH) of 1°, effectively removing microplastics from saline water. The anodized aluminum surface demonstrated exceptional anticorrosion capabilities in an aqueous solution with 3.5 wt% NaCl, further enhanced by superhydrophobic characteristics after 60 min of anodization. The functionalized surface exhibited superoleophilicity (0°) and superhydrophobicity (154°), successfully extracting microplastics from the NaCl aqueous solution with an efficiency exceeding 99%.

**Figure 28:**
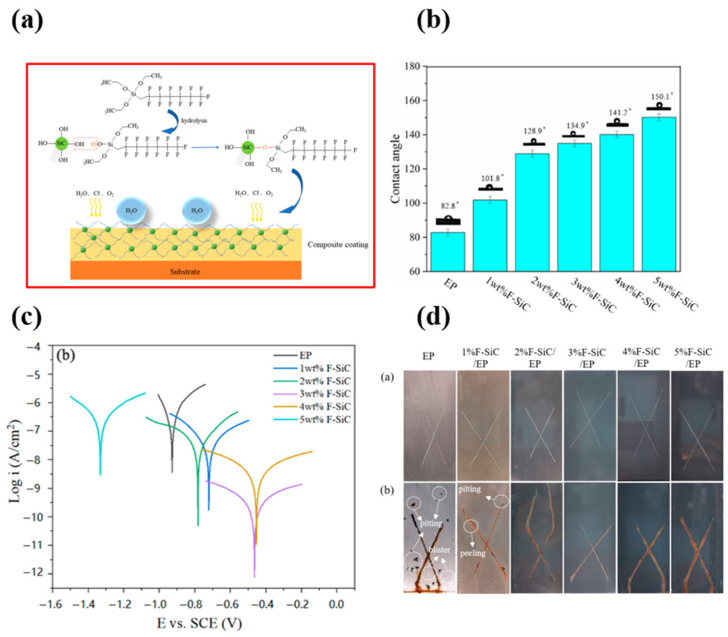
(**a**) Fabrication process for F−SiC and composite coating; (**b**) variation in the WCA of composite coatings with different F−SiC contents; (**c**) potential polarization test comparing different coatings; and (**d**) images depicting salt spray tests on different coatings (**a**) before the test and (**b**) after 240 h of test duration [210].

Similarly, Zhang et al. [210] employed a chemical grafting process to synthesize superhydrophobic SiC, where fluoroalkyl silane (FAS) was chemically bonded to the SiC surface. This superhydrophobic SiC was then integrated into epoxy resin (EP) to create a SiC/EP composite material. The organic molecule grafting on the SiC surface significantly improved the compatibility between nano-SiC and EP. The surface wettability of the coating changed with the addition of F–SiC; without F–SiC, the contact angle was less than 90°, but it increased significantly with F–SiC addition, as shown in Figure 28b. A superhydrophobic composite coating was achieved at 5 wt% F–SiC, reaching a static water contact angle of 150.1° with a roll-off angle of 5.5°. The corrosion resistance was optimal at a 3 wt% addition of F–SiC. The corrosion current of the composite coating was 2–3 orders of magnitude lower than that of the pure EP coating, signifying substantial improvement in corrosion resistance (Figure 28c). Additionally, a 240 h salt spray test evaluated various compositions of composite coatings (EP, 1–5 wt% F-SiC/EP), as depicted in Figure 28d. Results indicated that incorporating an appropriate amount of F-SiC enhanced the coating’s insulation capability, with optimal anti-corrosion performance observed in the F-SiC/EP composite coating at approximately 3 wt%. Table 7 summarizes previous research on anti-corrosion applications [168,211,212,213,214].

### 4.7. Anti-Bacterial Property

Bacterial colonization on surfaces poses significant challenges in various industries, healthcare settings, and everyday life. The proliferation of bacteria on surfaces can lead to the formation of biofilms, which are resilient and difficult to remove. In healthcare, bacterial contamination in hospitals can result in healthcare-associated infections (HAIs), posing a serious threat to patients with compromised immune systems. Similarly, in food processing and preparation areas, bacterial contamination can lead to foodborne illnesses. The growth of bacteria on surfaces is not only a hygiene concern but also impacts the durability and performance of materials, especially in outdoor settings where weathering and microbial attack can deteriorate surfaces over time. Consequently, finding effective solutions to mitigate bacterial growth on surfaces is crucial for maintaining public health, ensuring product integrity, and extending the lifespan of materials.

One promising approach to addressing the bacteria problem is the development of superhydrophobic anti-bacterial coatings. Superhydrophobic surfaces exhibit exceptional water repellency, preventing the adhesion and proliferation of bacteria and other microorganisms. These coatings combine the benefits of superhydrophobicity, which repels water and contaminants, with anti-bacterial properties to actively inhibit bacterial growth [215,216,217,218,219]. By incorporating antimicrobial agents or materials with inherent antibacterial properties into the coating formulations, researchers aim to create surfaces that not only repel water but also prevent bacterial colonization. This dual functionality makes superhydrophobic anti-bacterial coatings highly desirable for a wide range of applications, from healthcare settings and food processing facilities to everyday surfaces, ultimately contributing to improved public health, reduced maintenance costs, and enhanced material durability. Ye et al. [220] presented an environmentally friendly nanofibrillated cellulose-based multifunctional superhydrophobic coating (NMSC) through a silylation process involving cellulose, tetraethyl orthosilicate, and cetyl trimethoxysilane. Ethyl orthosilicate hydrolyzed into organosilicon, facilitated by ammonia water, imparted surface roughness to NFC. Hexadecyltrimethoxysilane hydrolysis lowered NFC surface energy. Post-silylation, the water contact angle significantly increased from 42° to 169°, showcasing remarkable hydrophobic, thermal stability, and self-cleaning properties. The NMSC demonstrated prolonged effectiveness against various fluids, including strong acid (pH 1) and alkali (pH 13), alcohols, alkanes, esters, and organic solvents. It maintained static contact angles above 155° during acid/alkali treatments, highlighting exceptional acid and alkali resistance. The NMSC exhibited antibacterial performance, attributed to its hydrophobic surface, which readily penetrated the phospholipid bilayers of bacteria, disrupting bacterial cell membranes and causing structural damage and cytoplasmic leakage.

**Figure 29:**
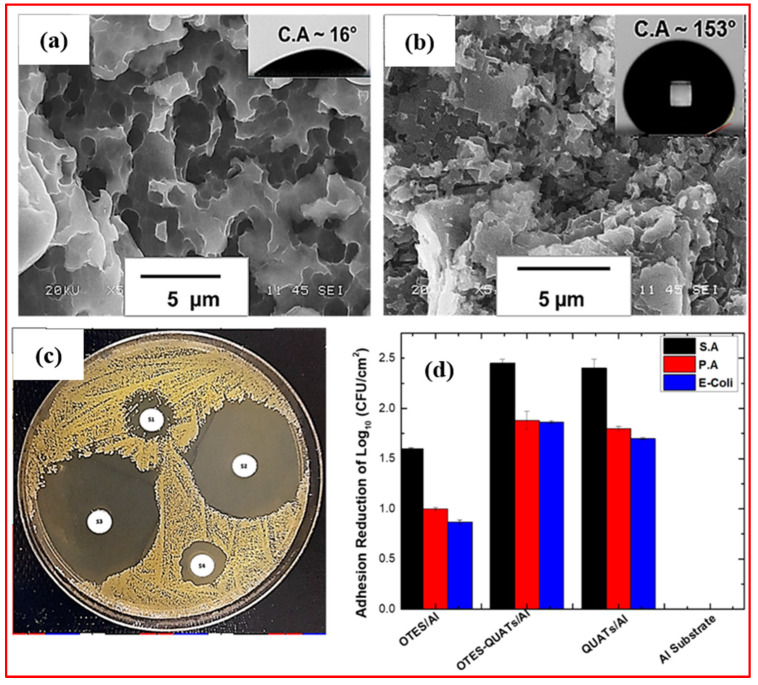
The surface morphology of the etched aluminum substrate (**a**) and OTES-QUATs/Al (**b**). Ethanoic solutions of OTES, OTES-QUATs, QUATs, and ethanol were subjected to disk diffusion assay against *Staphylococcus aureus* bacteria, with distinct regions labeled as S1, S2, S3, and S4, respectively (**c**). A graphical representation depicts the adhesion reduction of *Staphylococcus aureus*, *Pseudomonas aeruginosa*, and *Escherichia coli* on various surfaces, including OTES/Al, OTES-QUATs/Al, QUATs/Al, and etched Al substrate (**d**) [221].

Agbe et al. [221] illustrated a straightforward two-step procedure for creating a superhydrophobic and antibacterial aluminum surface. The process involves chemical etching in HCl, followed by immersion in an ethanolic solution of octyltriethoxysilane (OTES) and the addition of quaternary ammonium solution (QUATs) through drop-wise deposition. The chemical etching introduces micro- and nano-features with topological terraces, resulting in a surface root mean square (rms) roughness and contact angle (CA) of 6.2 ± 1.5 µm and 16° ± 0.2°, respectively. Post-modification, the OTES-QUATs/Al samples exhibited a reduced roughness of 5.8 ± 0.5 µm and an increased CA of 153° ± 3.7°, as depicted in Figure 29a–d. The antibacterial activity of the OTES-QUATs solution was noteworthy, displaying a zone of inhibition (ZOI) of 34 ± 1.6, 22 ± 1.4, and 25 ± 0.9 against *Staphylococcus aureus*, *Pseudomonas aeruginosa*, and *Escherichia coli*, respectively. The ZOI indicates the region around the antimicrobial agent where microbial growth is hindered, with a larger ZOI signifying more effective antimicrobial action. Furthermore, the OTES-QUATs-coated aluminum surface demonstrated excellent anti-biofouling properties, achieving a 99.9% reduction in bacterial adhesion for *Staphylococcus aureus*, 99% for *Pseudomonas aeruginosa*, and 99% for *E. coli* bacteria, attributed to the synergistic effects of low-energy OTES, micro/nano roughness, and the presence of QUATs, as depicted in Figure 29.

### 4.8. Water Harvesting

Water is a vital resource for all living organisms, and effective water harvesting plays a crucial role in sustainable water management, especially in regions dealing with water scarcity. Inspired by nature, harvesting water directly from the atmosphere emerges as a promising alternative, particularly in dry areas and regions with abundant fog. Nature provides various examples of efficient water harvesting mechanisms in different organisms and ecosystems, such as cactus spines, spider silk, the elytra of the Namib Desert beetle, and the inner wall of nepenthes [222,223,224,225].

The Namib Desert beetle exhibits remarkable water collection and self-transportation capabilities attributed to the surface energy gradient resulting from dual wettability on its elytra surface-wax-coated hydrophobic valleys and hydrophilic bumps. Fog-derived water droplets preferentially condense on hydrophilic bumps, and a surface energy gradient facilitates the transportation of water droplets from hydrophobic valleys to hydrophilic bumps. This nature-inspired mechanism demonstrates a potential solution for efficient water harvesting and transport [226,227,228,229,230].

Taking inspiration from the water-harvesting properties of the Namib Desert beetle, Zhai et al. [231] developed hydrophilic spots, each measuring 750 μm, on a superhydrophobic surface using a poly(acrylic acid) (PAA) water/2-propanol solution. In the patterned region, the advancing water contact angle was 144°, while the receding contact angle was 12°. These hydrophilic patterns emulate the wax-free areas on the Stenocara beetle’s back, where tiny water droplets from fog collect. As most droplets roll on the superhydrophobic regions, they eventually adhere to the hydrophilic patterns, forming larger water droplets. This mimics the water-capturing ability of the Stenocara beetle’s back, providing a method for capturing small water droplets and converting them into more substantial droplets.

**Figure 30:**
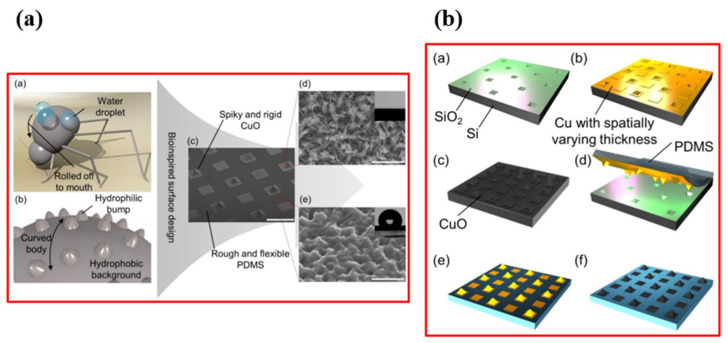
(**a**) A schematic overview of the fog-harvesting Namib Desert beetle and an SEM image of the fabricated flexible hybrid surface; (**b**) a schematic illustration of the fabrication process of the hybrid surface with a 3D superhydrophilic copper oxide pattern on a hydrophobic PDMS surface [232].

Similarly, Park et al. [232] explored fog harvesting using a flexible hybrid surface featuring a 3D superhydrophilic copper oxide (CuO) pattern on a hydrophobic, rough polydimethylsiloxane (PDMS) background. This design drew inspiration from the bumps found on the curved dorsal surface of Namib desert beetles, as shown in Figure 30a. The process involved transferring a copper (Cu) layer from a silicon (Si) or glass donor substrate to a PDMS receiving substrate, molded on, and then peeled from the donor substrate. Following the transfer, the Cu layer was patterned and oxidized, forming a superhydrophilic CuO pattern on the hydrophobic, rough PDMS substrate. Consequently, the transferred and oxidized Cu layer on the PDMS substrate could exhibit 2D or 3D patterns, depending on the initial morphology of the donor substrate, as depicted in Figure 30b. Evaluation of the water collection rates for the prepared surface indicated that the 3D bumpy structures on a curved surface showed over 16 times higher water collection rates than the flat 2D hybrid surface when subjected to a fog stream.

Recently, Choi et al. [233] presented a hierarchically structured surface with microgrooved patterns achieved through FFF-type 3D printing. The design aimed to replicate the morphology of cactus spines and the dual wettability observed in the Namib Desert beetle. Microgrooved patterns resembling those of cactus spines were incorporated onto the cactus-shaped PDMS surface using the staircase effect inherent in FFF-type 3D printing. To mimic the dual wettability of Namib Desert beetles, a partial Pt deposition method was introduced, employing a paraffin wax-based masking method for mass-producing complex morphologies. Capitalizing on the surface energy gradient, the hydrophobic region could condense more water droplets than the hydrophilic region, yet the condensed water in the hydrophobic region could autonomously transport itself to the hydrophilic region. 

Additionally, the cactus spine morphology facilitated the self-transportation of condensed water droplets from the tip to the base region using the Laplace pressure gradient. The resulting surface demonstrated superior fog harvesting performance (average weight of 7.85 g for 10 min), benefiting from the synergistic interplay between the Laplace pressure gradient and surface energy gradient.

These findings offer valuable insights for the future design and deployment of hybrid superhydrophobic/superhydrophilic surfaces for cost-efficient atmospheric water harvesting, supporting effective fog harvesting to obtain freshwater under harsh conditions, including dry and polluted water environments.

### 4.9. Medical Industry

**Figure 31:**
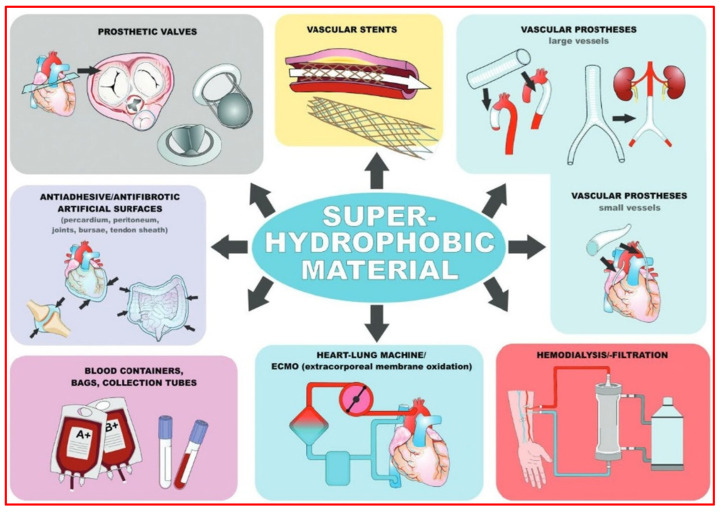
Superhydrophobic coatings for blood-repellent applications in the medical field [29].

Superhydrophobic polymeric nanocoatings find diverse applications in the medical field, including dentistry, self-cleaning, and drug delivery [234]. Sun et al. [235] innovatively designed a cardiopulmonary bypass tube with superior blood repellency and superhydrophobicity. The results revealed that the superhydrophobic-treated tube clotted in 36 min in terms of coagulation time, biotoxicity, platelet adsorption, and protein, in contrast to a clinical Bioline heparin-coated tube (21 min) and a bare PVC tube (14 min). The superhydrophobic-treated tube demonstrated a remarkable 157% increase in clotting time compared to the bare PVC tube. Additionally, protein and platelet adsorption on the superhydrophobic-treated tube witnessed reductions of 32% and 74%, respectively. Figure 31 illustrates the application of superhydrophobic surfaces in medical devices to prevent blood stickiness [29]. In addressing microleakage in dental composite restorations, superhydrophobic coatings were developed using photo-crosslinked polyurethane (PU) and SiO_2_ nanoparticles functionalized with organic fluoro groups (F-SiO_2_ NPs). The study revealed that a low concentration ratio of PU/F-SiO_2_ (1:3) in superhydrophobic coatings exhibited desirable attributes, including good transparency, a high contact angle (160.1°), a low sliding angle (<1°), and an excellent hierarchical papillae structure [236].

While the potential applications are promising, challenges such as long-term durability, scalability, and cost-effectiveness need to be addressed for successful integration into various industries. Ongoing research in materials science is likely to uncover new possibilities, and staying abreast of the latest developments in the field is essential for a comprehensive understanding of the future of superhydrophobic materials.

## 5. Challenges and Constraints in Superhydrophobic Surfaces

While superhydrophobic surfaces present promising opportunities across various applications, they also encounter several challenges that need to be addressed for their widespread adoption and practical implementation. These challenges encompass both fundamental aspects and practical considerations and are crucial for advancing the field and realizing the full potential of superhydrophobic materials. Several key challenges are worth highlighting:

### 5.1. Durability and Stability

To enhance the practical usability of artificial superhydrophobic coatings, they must exhibit strong adhesion as well as robust mechanical and chemical stability. Generally, the long-term stability and durability of superhydrophobic surfaces pose significant challenges. External factors such as abrasion, chemical exposure, and environmental conditions can impact the integrity of these surfaces over time. Developing robust materials and fabrication techniques that withstand prolonged usage remains a critical challenge.

### 5.2. Scalability and Cost-Effectiveness

Many fabrication methods for superhydrophobic surfaces are complex and may not be easily scalable for large-scale industrial applications. The cost of materials and manufacturing processes can pose economic challenges, hindering the widespread adoption of superhydrophobic technologies. Ensuring cost-effectiveness and scalability while maintaining the desired properties is essential for widespread adoption in various industries.

### 5.3. Biocompatibility and Health Concerns

In biomedical applications, ensuring the biocompatibility of superhydrophobic materials emerges as a critical consideration. Biocompatibility involves a comprehensive assessment of the interactions between living organisms and these superhydrophobic materials. Particularly in medical applications, such as implants or medical devices, it becomes imperative to scrutinize how these superhydrophobic surfaces interact with biological tissues and fluids. The incorporation of certain materials in superhydrophobic coatings, especially those containing nanoparticles or specific chemical compounds, raises potential health concerns. The release of nanoparticles into the body may lead to long-term health impacts. Achieving a delicate equilibrium between attaining superhydrophobic properties and ensuring biocompatibility stands as a crucial factor for the responsible and successful integration of these surfaces across various fields without compromising the well-being of both humans and the environment.

### 5.4. Multifunctional Superhydrophobic Coating/Surfaces

**Figure 32:**
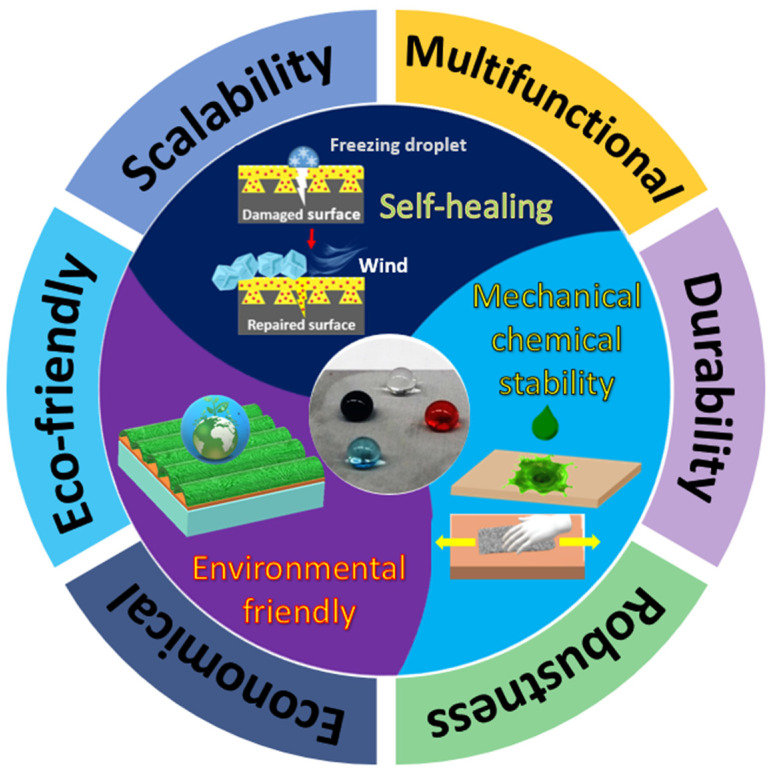
Bio-inspired artificial superhydrophobic surfaces with multifunctional properties.

Multifunctional superhydrophobic surfaces represent a cutting-edge area of research that aims to develop materials with a range of enhanced properties beyond water repellency. These surfaces combine superhydrophobicity with diverse functionalities, broadening their potential applications across various fields, as shown in Figure 32. Despite the advantages of multifunctional superhydrophobic surfaces in many applications, they do present certain limitations. For instance, achieving robust self-cleaning capabilities may compromise other aspects, such as mechanical durability or chemical stability. Additionally, the integration of multifunctionality often involves complex surface structures and coatings, making fabrication and scalability more intricate. Concerns also arise regarding the durability of these surfaces under harsh environmental conditions or during prolonged use. Addressing these limitations is crucial to propelling the practical and widespread adoption of superhydrophobic surfaces across diverse applications.

### 5.5. Environmental Impact

The environmental implications of superhydrophobic materials, particularly concerning their production and the potential release of nanostructures into the environment, have raised significant concerns. It is essential to develop environmentally friendly fabrication methods and gain a comprehensive understanding of the ecological consequences to ensure responsible use. A key concern is the use of certain chemicals and materials in the manufacturing of superhydrophobic coatings. Reports have confirmed that some solvent-based ‘water-repellent’ coatings pose health risks, causing harm to the lungs, kidneys, nerves, teeth, and promoting bone decay. The utilization of fluorine-based coatings, particularly in fine spray forms, increases the risk of inhalation and irritation to the eyes and nose.

Over the last 30 years, several hundred cases of serious respiratory inflammation have been reported among consumers using hydrophobic coating ‘spray-on’ products in Europe and the USA. Some countries have banned the use of fluorinated compounds due to their adverse side effects; for instance, polyfluorooctyl-triethoxysilane (1H,1H,2H,2H-perfluorooctyl triethoxysilane) is banned in Denmark, with restricted use in Canada. Therefore, sustainable and eco-friendly approaches to material selection, manufacturing processes, and end-of-life management are crucial for minimizing any adverse effects on the environment.

As researchers and engineers continue to investigate these challenges, overcoming them will unlock the full potential of superhydrophobic surfaces and drive innovation across diverse fields. Overcoming these obstacles will not only refine the fundamental understanding of superhydrophobicity but also lay the foundation for practical, real-world applications with enduring significance.

## 6. Progress in Wetting Research through AI and Machine Learning

The incorporation of artificial intelligence (AI) algorithms into materials design is transforming the landscape of materials engineering. This is attributed to their capability to forecast material properties, create novel materials with improved characteristics, and unveil previously unrecognized mechanisms, surpassing traditional intuition in the process. Despite this, there has been limited in-depth exploration into the AI-assisted design of material textures.

The swift progress in artificial intelligence (AI) and machine learning (ML) presents significant possibilities for transforming and accelerating the laborious and expensive material development process. Over the past few decades, AI and ML have inaugurated a fresh era in materials science, employing computer algorithms to assist in exploration, comprehension, experimentation, modeling, and simulation [237,238]. Collaborating with human ingenuity and creativity, these algorithms play a crucial role in uncovering and enhancing innovative materials for upcoming technologies.

Lu et al. [239] introduced a graph-focused deep learning technique specifically designed to capture the intricate nuances inherent in spider web architectures. The utilization of this technique extends beyond understanding these complexities, serving the dual purpose of facilitating the generation of a diverse range of innovative bioinspired structural designs. The authors have laid the foundation for a groundbreaking framework for the generation of spider webs, delving into the realm of bioinspired design guided by rigorous principles. Not confined to spider web emulation, this method demonstrates versatility in addressing various heterogeneous hierarchical structures, spanning a wide spectrum of architected materials. Positioned as a valuable toolset in the domain of generative AI for material applications, this approach bridges the gap between theoretical exploration and the practical actualization of designs, offering profound insights into biology and diverse design possibilities.

Traditionally, the study and design of bioinspired structures have relied on empirical, extensive, and time-consuming top-down strategies devoid of AI algorithms. Scientists painstakingly observe and analyze natural organisms to discern the fundamental principles and structures contributing to their exceptional properties [240,241,242,243]. Designing and fabricating materials using advanced manufacturing techniques, conducting experiments to validate performance, and refining designs based on outcomes are integral to this resource-intensive and time-consuming approach, often requiring significant trial and error.

To overcome these challenges and expedite the bioinspired material design process, the integration of AI algorithms offers significant potential to enhance efficiency and effectiveness. Leveraging supervised AI, researchers can expedite material development by swiftly exploring a vast design space, reducing the pool of potential solutions, and pinpointing optimal material compositions and structures for a chosen application [244]. However, it is crucial to note that supervised learning demands high-quality data with accurate labeling about behaviors and characteristics to function correctly. Yu et al. [245] employed reinforcement learning to achieve a refined design within an unfamiliar design space, developing a model capable of learning a biological design strategy through multiple training iterations. In their methodology, they utilized a finite element method (FEM) to compute mechanical properties, treating them as “reward values” for the algorithm to produce materials with enhanced fracture toughness. To ensure highly optimized solutions and bolster confidence, the AI initially analyzed a small set of systems, gradually increasing the size with each successful convergence of results. This iterative approach aimed to minimize calculation time for obtaining the optimal design. Throughout the training process, the model acquired the capability to understand the biological design strategy, enabling its extension to more complex structural optimization problems by incorporating different mechanical properties as reward values. This innovative design framework, adaptable to modifications in system intricacy or the inclusion of additional variables as rewards, demonstrates versatile utility across various applications.

In contrast, Lantada et al. [246] used artificial neural networks (ANNs) to forecast the wettability of bioinspired and biological structures according to their hierarchical surface characteristics. The ANN model was trained using a vast collection of bio-surfaces that the authors had assembled with well-known wettability characteristics. New, distinct topographies were then added in order to assess the algorithm’s effectiveness. The wettability findings were compared with those predicted by the AI model once the actual structures were constructed. The convergence of results demonstrated how well artificial intelligence (AI) may help discover new bio-interfaces with hierarchical tribology, allowing for fine control over wettability. This method may take into account a large number of factors that would provide several controlled characteristics and behaviors, increasing its flexibility for particular applications.

Finally, using AI can save on expenses related to developing new materials. Indeed, less intensive laboratory experimentation is required when the design process is streamlined. Gu et al. [247] stated the mechanical characteristics of 100,000 microstructures with respect to computing cost. The procedure took about five days when using FEM, but with their designed ML technique, the same amount of data could be solved in less than a minute during the prediction phase and between 30 s and 10 h during the training phase.

In this way, scientists may focus on the most promising ideas and expedite the material design process, increasing its effectiveness and economy, by utilizing AI’s computing capability. Even though this trip is still in its early stages, we are convinced that AI will support materials scientists’ research rather than work against it. This will greatly broaden the field’s perspectives, creating new opportunities and quickening the development of material design.

## 7. Practical Implications of the Present Review

This article provides a comprehensive review of superhydrophobic surfaces, encompassing fundamental understanding, natural occurrences, design principles of bio-inspired coatings, potential applications, challenges, and future research directions. The practical implications of this review are multifaceted, offering valuable insights for various stakeholders:

1. Innovative material design: This review identifies key principles inspired by nature that can guide the development of advanced superhydrophobic nano-coating materials. Researchers and industry professionals can leverage these insights for innovative material design, paving the way for the creation of superior products with enhanced functionalities.
2. Functional applications: By drawing inspiration from natural structures, this review proposes practical applications across various domains. These insights could result in the development of coatings customized for specific functions such as self-cleaning, anti-fouling, anti-icing, and more. Implementing nature-inspired coatings with tailored functionalities could significantly impact industries dealing with surface protection and maintenance challenges, ultimately reducing maintenance expenses and prolonging the life of diverse substrates.
3. Environmental sustainability: The exploration of environmentally friendly coatings inspired by nature aligns with global efforts for sustainable practices. Understanding the principles of natural structures allows the development of coatings that exhibit superior performance while contributing to eco-friendly coating technologies, reducing the environmental impact of conventional materials.
4. Advancements in biomedical coatings: This review highlights the relevance of nature-inspired coatings in biomedical applications, offering potential advancements in medical devices, implants, and drug delivery systems. Coatings designed to interact favorably with biological systems can lead to improved biocompatibility and reduced adverse effects, fostering progress in healthcare technologies.
5. Commercialization opportunities: Synthesizing the state-of-the-art in nature-inspired nano-coating materials, this review identifies promising avenues for commercialization and technology transfer. These insights inform the development of marketable products, driving growth in the coatings industry and contributing to economic development.
6. In conclusion, the practical implications of this review extend to fostering innovation, tailoring functionalities in coatings, promoting sustainability, advancing biomedical applications, identifying commercialization opportunities, and facilitating interdisciplinary collaboration. The comprehensive insights provided pave the way for real-world applications and transformative advancements in the field of nano-coating materials.

## 8. Conclusions

In conclusion, this review highlights the significant advancements in the field of superhydrophobic surfaces and their diverse applications. From the fundamental principles governing their unique wetting properties to the various fabrication techniques employed, a comprehensive understanding of these surfaces has been presented. The exploration of natural examples, such as lotus leaves and butterfly wings, has inspired innovative designs for artificial superhydrophobic surfaces. The applications across different industries, including self-cleaning materials, oil–water separation, and anti-icing technologies, underscore the immense potential of superhydrophobic surfaces in addressing real-world challenges. Moreover, this review emphasizes the importance of continued research to overcome challenges such as durability and scalability and to unlock new opportunities for this technology. As we look toward the future, the development and integration of superhydrophobic surfaces are composed to make a transformative impact, offering sustainable and efficient solutions in various fields.

## Figures and Tables

**Table 1:** Superhydrophobic surfaces present in nature.

Natural Surface	WCA	Properties	References
Rice leaf	164°	Superhydrophobic, self-cleaning, antifouling, and low drag	Bixler et al. (2014) [14]
Mosquito compound eyes	155°	Superhydrophobic and anti-fog	Gao et al. (2007) [16]
Butterfly wings	152 ± 1.7°	Superhydrophobic and self-cleaning	Zheng et al. (2007) [17]
Gecko foot	150°	Superhydrophobic and anti-adhesion	Stark et al. (2016) [19]
Water strider legs	167.6 ± 4.4°	Superhydrophobic and anti-adhesion	Gao et al. (2004) [20]
Lotus leaf	>150°	Superhydrophobic and self-cleaning	Barthlott et al. (1997) [24]
Indian cress	180°	Superhydrophobic and self-cleaning	Otten et al. (2004) [25]
Shark skin	160°	Superhydrophobic, self-cleaning, and anti-fouling	Liu et al. (2012) [26]
Desert beetle	>150°	Superhydrophobic and fog-collection behavior	Kostal et al. (2018) [27]
Rose petal	154.6°	Superhydrophobic and high surface adhesion	Feng et al. (2008) [28]

**Table 2:** Representative review articles on superhydrophobic coatings in recent years (2020–2024).

S. No.	Title	Journal	Publication Date	Features	Ref.
1	Recent advances in superhydrophobic and antibacterial coatings for biomedical Materials	*Coatings*	October 2022	Summarized development trends in medical device coatings, focusing on superhydrophobic and antimicrobial coatings. Addressed potential applications and challenges in the commercial adoption of antimicrobial coatings.	Wang et al. [29]
2	3D-printed biomimetic structures for energy and environmental applications	*DeCarbon*	March 2024	An overview of the current state of 3D-printed biomimetic structures and their applications in energy and environment.	Li et al. [30]
3	Recent progress on transparent and self-cleaning surfaces by superhydrophobic coatings deposition to optimize the cleaning process of solar panels	*Solar Energy Materials and Solar Cells*	August 2023	An overview of the latest studies on self-cleaning superhydrophobic coatings for solar energy applications.	Nomeir et al. [31]
4	Bioinspired marine antifouling coatings: antifouling mechanisms, design strategies and application feasibility studies	*European Polymer Journal*	May 2023	Summarization of the design strategy and development trend of bionic marine antifouling coatings, evaluates the antifouling performance, antifouling mechanism, and antifouling effect of the coatings.	Li et al. [32]
5	Recent advances in bioinspired sustainable sensing technologies	*Nano-Structures & Nano-Objects*	April 2023	A comprehensive overview concerning the innovative approach for identifying, recognizing and showcasing the current advancements and key milestones accomplished in biosensing technologies based on bioinspired/bioderived materials.	Mishra et al. [33]
6	Recent advances in bio-inspired multifunctional coatings for corrosion protection	*Progress in Organic Coatings*	July 2022	An overview of research findings on bioinspired organic/inorganic superhydrophobic and slippery coatings for the corrosion protection of metal substrates.	George et al. [34]
7	Icephobic/anti-icing properties of superhydrophobic surfaces	*Advances in Colloid and Interface Science*	June 2022	The research progress of superhydrophobic materials on icephobictiy in recent years is reviewed from the aspects of ice formation and propagation.	Huang et al. [35]
8	Bioinspired and green synthesis of nanoparticles from plant extracts with antiviral and antimicrobial properties: A critical review	*Journal of Saudi Chemical Society*	September 2021	The advancement in green synthesis of nanoparticles using natural compounds such as plant extracts, fruit juices, and other relevant sources have been highlighted. A deep insight into antiviral and antimicrobial activities of these nanoparticles provided.	Naikoo et al. [36]
9	Superhydrophobic and superoleophilic membranes for oil–water separation application: A comprehensive review	*Materials & Design*	June 2021	A comprehensive review of the SHSO membranes, fabrication and characterization methods, the advantages and disadvantages of the fabrication techniques, current status and prospects of SHSO surfaces, and potential future research directions.	Rasouli et al. [37]
10	Bioinspired materials for water-harvesting: focusing on microstructure designs and the improvement of sustainability	*Materials Advances*	November 2020	Comprehensive insights into the bioinspired water-harvesting materials, focusing on the microstructure designs and improvements of sustainability	Zhang et al. [38]
11	Bioinspired polymers for lubrication and wear resistance	*Progress in Polymer Science*	November 2020	Bioinspired lubrication using novel polymeric structures, which has led to producing a myriad of new systems with effective and sustainable antifriction and wear resistant properties.	Adibnia et al. [39]
12	Nature-inspired nano-coating materials: drawing inspiration from nature for enhanced functionality	*Micromachines*	2024	Comprehensive overview of the current state of superhydrophobic research. This review aims to guide future investigations and inspire innovations in the development and utilization of these fascinating surfaces.	Present work

**Table 3:** Advantages and disadvantages of the superhydrophobic preparation process during practical production.

Fabrication Method	Principle	Advantages	Disadvantages
Chemical etching	Dissolution of surface layers using etchant solutions	Simple, fast, cost-efficient, and scalable Create durable complex micro/nanostructures	Limited control over surface morphology Applicable to metals and alloys mainly Use of toxic solutions Poor film uniformity
Lithography	Involves the transfer of a pattern from a mask to a substrate using light, radiation, or other forms of energy	Simple and relatively fast Precise control over surface features Ability to create complex patterns Environmentally friendly Applicable to wide range of materials Reusability of templates	High initial equipment and setup costs, not scalable Multi-step process; requires a flat substrate May require cleanroom Potential waste generation from resist and developer chemicals
Template	Using templates to create structured surfaces	Low-equipment demand and harmless Durable, well-defined structures Scalable	Template removal may be challenging Limited flexibility
Plasma	Alteration of surface properties using plasma	Simple, high aspect ratio structures Applicable to a wide range of materials Enhanced adhesion and versatile	Decrease mechanical properties Costly; potential toxic gas formation Limited control over surface roughness
Sol–gel	Condensation polymerizationreactions under colloidal liquidsystems	Cost-efficient and applicable to various substrates Tunability of size and morphology of particles in film, Scalable Synthesis at normal temperature Provides high quality films; less deterioration	Slow process; typically requires multiple processing steps Limited durability May require multiple steps
Hydrothermal	Dissolution; recrystallisation processes at high temperature and pressure	Simple, cost-effective process Environmentally friendly Durable and scalable Allows for the growth of hierarchical structures	High equipment requirements Limited to certain materials Requires precise control of reaction parameters
Electrospinning	Droplet spraying and stretching in electric field	Inexpensive and environmental-friendly High superhydrophobic performance High surface area; fine structures Film homogeneity	Non-durable; low fiber strength Limited control of porosity; relatively slow Limited to polymer fibers Requires specialized equipment Limited scalability
Chemical vapor deposition	Vapor-phase deposition of precursor chemicals	Uniform coating; residue-free Film homogeneity Control over coating thickness and composition	Complex equipment and high cost Limited to specific substrates Requires high temperatures and controlled environments
Electrochemical deposition	Deposition of material through electrochemical reaction	Cost-efficient and scalable Uniform coating; control over surface morphology Tunability of texture morphology	Limited material compatibility Limited durability; complex process optimization Possible toxicity
Layer-by-layer	Inter-particle electrostatic interaction	Precise control of layer thickness Cost-efficient and versatile Multifunctionality; tunable surface properties	Time-consuming, complex processes May not be suitable for large surfaces Limited scalability; potential for delamination Sensitivity to environmental conditions

**Table 4:** Self-cleaning performance of some coated materials on different substrate.

Fabrication Technique	Coating Material	Substrate	Findings	Reference
Modification with HDTMS	Zn-MOF	Cotton fabric	The fabric coating exhibited a WCA of 160° with a hysteresis of 7°, showcasing stable superhydrophobic and self-cleaning characteristics.	[158]
Solvothermal and chemical modification method	Nano zinc sulfide (ZnS)	Zinc	Superhydrophobic ZnS coating has excellent chemical and physical self-cleaning properties.	[159]
Atmospheric pressure plasma polymerization	Hexamethyldisiloxane (HMDSO)	Glass	The thin films on a glass exhibit outstanding superhydrophobic and self-cleaning properties, featuring a WCA of 165° and an SA of about 2°.	[160]
One-step sol. immersion process in Mn (II) aqueous solution and post-modification by stearic acid	Manganese dioxide (MnO_2_) microspheres	Mg alloy	Fabricated superhydrophobic magnesium alloy exhibits remarkable self-cleaning properties in both oil and air.	[161]
Dip coating method	Titanium dioxide nanomaterial	Glass	Fabricated superhydrophobic glass shows an excellent potential for self-cleaning action against contaminants.	[162]

**Table 5:** Anti-icing performance of some coated material on different substrate.

Fabrication Technique	Coating Material	Substrate	Findings	Ref.
Laser ablation, followed by modification with HDTMS	Hexadecyltrimethoxysilane (HDTMS)	45 steel	Superior anti-/de-icing properties; ice on surface thaws in 60 s under 0.5 sun illumination. Exhibits a 2547s longer freezing delay time and at least ∼3 times lower de-icing force than steel substrate.	[168]
Combination of simple chemical etching and anodization, along with modification with poly(dimethylsiloxane) (PDMS)	Silicone oil-infused PDMS (SOIP) coating	Aluminum (Al)	SOIP coating displayed lower ice-adhesion strength of 22 ± 5 kPa compared to superhydrophobic coatings. Surface has minimal ice-adhesion (108 kPa) after 20 icing/de-icing cycles, and long-term icephobicity (55 ± 13 kPa) after 4 months of exposure to ambient environment.	[169]
Simple spraying and curing process	Nanosilica co-modified with fluoroalkyl silane and aminosilane	Polyurethane	Excellent anti-icing efficiency Delaying water-freezing (700 s) at −15°. Ice-adhesion strength lower than 13 KPa after 25 icing-deicing cycles	[170]
Chemical etching and anodization, later modified with PDMS via thermal vapor deposition.	PDMS	Aluminum	WCA of fabricated surface more than 160 °C. Superhydrophobic surface showed exceptional anti-icing properties Ice formation on superhydrophobic surface was delayed by 45 and 80 min at −10 °C and −5 °C, respectively, at a relative humidity of 80% ± 5%.	[171]
Crystal growth method	Hollow micro-/nano-structured ZnO (HMN)	Silicon	HMN ensures a significant thermal resistance between the base and liquid droplet, resulting in enduring anti-icing performance at lower temperatures.	[172]

**Table 6:** Oil–water separation performance of some coated material on different substrates.

Fabrication Technique	Coating Material	Substrate	Findings	Ref.
Modification with HDTMS	Zn-MOF	Cotton fabric	Superhydrophobic surface showed exceptional efficiency of 93, 95, 97, 98%, and 100% in separating engine oil, crude oil, n-hexane, chloroform from water, and viscose oils (sunflower and coconut), and 90% for stabilized emulsion.	[158]
Three simple processes: (1) synthesis of MOF-5 nanoparticles; (2) modification using PFOTS; and (3) dip-coating method	Zinc-based metal–organic frameworks (MOF-5)	Sponge	Continuous separation of a variety of oil–organic solvent–water mixtures with a separation efficiency >98%.	[201]
Plasma polymerization	Hexamethyldisiloxane (HMDSO)	Fabric and offset printing paper	Superhydrophobic coating has an excellent separation efficiency with the oil contact angle (OCA) of about 0° under the optimal working conditions.	[160]
Combining chemical etching and hydrothermal processes	PDMS	Aluminum	Superhydrophobic/superoleophilic mesh shows high separation efficiency (94%) and outstanding reusability.	[202]
Dip-coating method	MWCNTs/ZnO composite	Copper mesh	Superhydrophobic and superoleophilic mesh exhibits remarkable reusability and excellent separation efficiency of over 95% for various oils.	[203]

**Table 7:** Anti-corrosion performance of some coated materials on different substrates.

Fabrication Technique	Coating Material	Substrate	Findings	Ref.
Pulse laser ablation, followed by modification with HDTMS	Hexadecyltrimethoxysilane (HDTMS)	45 steel	Surface shows superior corrosion resistance compared to steel, with 73.81 times higher charge transfer resistance and 64.78 times lower corrosion current density.	[168]
Combination of chemical etching with hydrothermal process, followed by PDMS coating via a simple vapor deposition method	Polydimethylsiloxane (PDMS)	Aluminum alloy	Corrosion resistance of bare surface is significantly enhanced by a magnitude of three after becoming superhydrophobic surface.	[211]
Hydrothermal method, with modification by sodium laurate (SL) and sodium dodecylbenzene sulfonate (SDBS).	MgAl-LDH laminates	AZ31 alloy	In a 3.5 wt.% NaCl solution, functional coatings demonstrated remarkable anti-corrosion performance.	[212]
Sandblasting and acid treatment, followed by electrodeposition and hydrophobic modification.	Ni-W-TiO_2_ coating	Steel	Superhydrophobic composite coating can attain a corrosion inhibition rate of 99.63% in 3.5% NaCl solution and at 25 °C.	[213]
Eco-friendly green method	Lauric acid	Concrete	Superhydrophobic concrete (WCA > 153° and WSA < 10°) has better corrosion resistance to internal rebars than ordinary concrete.	[214]

## Data Availability

Not applicable.
